# Supplementary material for: Allosteric regulation and crystallographic fragment screening of SARS-CoV-2 NSP15 endoribonuclease
Source: Nucleic Acids Res. 2023 Apr 28;51(10):5255–70. doi: 10.1093/nar/gkad314 (PMC10250223; doi:10.1093/nar/gkad314)
Supplement: gkad314_Supplemental_Files [file gkad314_supplemental_files.zip › Supplementary datasets.zip!/231/Refine_8/01-BUSTER/Cycle-2/LIST.html]

Refining

**BUSTER 2.X**: A suite of programs for macromolecular structure refinement by maximum-likelihood and Bayesian methods  
Copyright © 1992-2020 Global Phasing Ltd.  
All rights reserved.  
Authors: G. Bricogne, E. Blanc, M. Brandl, C. Flensburg, P. Keller, W. Paciorek, P. Roversi, O. Smart, C. Vonrhein, T. Womack  

Please reference use of the software as:

- BUSTER-TNT 2.X, Global Phasing Ltd, Sheraton House, Cambridge CB3 0AX, UK

Version: 2.10.3 for linux64-ifort made on May 20 2020 at 01:14:34  
Licenced to: Diamond Light Source, Scientific Software  

```
---


### Parsing of the BUSTER cardfile

TITLE : autoBUSTER refinement

   Cell information

   Symmetry information

 Using CCP4 X-ray form factors.

   Missing structure chemical composition

 Using ATOM block for chemical composition.


   Shell information

### Parsing of the TNT cardfile


---


### Reading observations file

 Number of reflexions in input:  85622

      Number of accepted reflexions:  81660

      Number of rejected reflexions:      0

   Reflexions in test set:            4077

   Reflexions in working set:        77583


---


### 1. Generating initial model

 2.10 * Shannon sampling rate. NX NY NZ:   360  360  240


  Effective Shannon rates are:    2.50    2.50    2.26


   Allocated BUFFER of size:    119.312 Mb.

   Initial Geometry Information


 No missing atoms declared


   Summary of model volumes

 No missing atoms declared: no prior computed


 Computing bulk solvent model scattering


 NCSMASK PDB radius for solvent mask:     2.15 Angstrom

 running PDBSET (setting cell and spacegroup P1) ... done
 running NCSMASK [1] (creating mask, SMOOTH 0.921083) ... done
 running MAPDUMP (to check if reduction to unit cell is needed) ... done
 running MAPMASK (converting mask to map) ... done

   Generation of babslv by theta-filter

   Electron density in frg mask:  0.420 Å-1

 FOBS  put on rough absolute scale by Wilson plot :
           K        =  0.37640E+02
           B        =    -11.782

 

### Cycle 0001: MaxLik scaling and refinement


   MaxLik Scaling Cycle 0001

   Wilson plot

   Statistics in resolution bins just after scaling
```

|  |  |
| --- | --- |
|  | - Rxpct factors plot- Rxpct factors table- <Log-Lik Gain>- Ln Sigma A- Correlation Coefficients |

```
   <Log-Likelihood> of null-hypothesis

Structure Factor Amplitudes


   Average Figure of Merit

 Switching off refinement of overall temperature factor


 Added B =     0.3210 Å2 to all atoms in fragment


 Added B =     0.3210 Å2 to B_SOLV

```
gelly refinement module
 Authors (2004-2009) O. S. Smart, C. Flensburg, W. Paciorek, C. Vonrhein, 
                     M. Brandl, T. O. Womack and G. Bricogne
         (2010-2011) O. S. Smart, C. Flensburg, W. Paciorek, C. Vonrhein, 
                     T. O. Womack and G. Bricogne
         (2012-2014) O. S. Smart, C. Flensburg, W. Paciorek, C. Vonrhein, 
                     and G. Bricogne
         (2015-2020) G. Bricogne, C. Flensburg, W. Paciorek, C. Vonrhein
 Copyright © 2004-2020 Global Phasing Ltd. 
 All rights reserved.
 gelly module compilation date May 20 2020 01:11:20
 
 gelly uses routines from TNT geometry. References: 
     Tronrud, D. E., Ten Eyck, L. F., Matthews, B. W. (1987)
     "An Efficient General-Purpose Least-Squares Refinement Program 
     for Macromolecular Structures". Acta Crystallogr A, 43:489-501.
     Tronrud, D. E. (1997). "The TNT Refinement Package", 
     in Macromolecular Crystallography, Part B, Eds Charlie Carter 
     and Robert Sweet, Methods in Enzymology, 277:306-319.
 wildcard matching function by Julian Robichaux http://www.nsftools.com

 No -random_seed flag found. Random number stream initialised using:
2147483562
2147483398

 Control flags set to:  -autoncs -hydrogens_input_occ2zero \
                     -hydrogens_output_occ2heavy -screen \
                     100 -screen_sigma \
                     3.0 -keeppdb \
                     1000000 -taomonitor_verbose \
                     2 
 

 ############### Starting setup via TNT routines

 Setting up tnt routines including license check:

                   TNT Stereochemistry Program
 Version: 5.22.3.0
 Licence for Diamond Light Source, Scientific Software is valid
 
 Using TNT geometry routines to load the tnt control file: gelly.tnt
 Using TNT routines to load initial coordinates from file fragment.cor
 Expecting 6138 atoms

 Found     2195 "NOTE BUSTER_RESIDUE_ATOM_DATA" cards
 Have copied information for    6138 atoms from TNT to gelly's store 
 
 The TNT WEIGHT cards used by this run:
 -----WEIGHT       BOND     2.0
 -----WEIGHT       ANGLE    2.0
 -----WEIGHT       IMPROPER 0.0
 -----WEIGHT       TORSION  2.0
 -----WEIGHT       PSEUDO   0.0
 -----WEIGHT       TRIGONAL 2.0
 -----WEIGHT       PLANE    5.0
 -----WEIGHT       CONTACT  5.0
 -----WEIGHT       BCORREL  10.0
 -----WEIGHT       CHIRAL   5.0
 -----WEIGHT       NCS      50.0
 -----WEIGHT       IDEAL    4.0
 -----WEIGHT       RFACTOR  2.13
 
 Dictionary versions picked up from NOTE BUSTER_RESTRAINT_DICT cards as: 
 ------ protgeo_eh99.dat (V1.6.4.17) 20191001  STANDARD AMINO ACID DICTIONARY. BONDS AND ANGLES FROM ENGH AND HUBER EH99. OTHER VALUES BASED ON PREVIOUS TNT OR TAKEN FROM CCP4. INCLUDES HYDROGEN ATOMS.
 ------ exoticaa.dat (V1.3.4.15) 20200327  COLLECTION OF NON-STANDARD AMINO ACIDS, MAINLY EH91 WITHOUT IDEAL DISTANCE INFO
 ------ nuclgeo.dat (V1.13.4.8) 20190909
 ------ bcorrel.dat (V1.15.10.2) 20190616
 ------ contact.dat (V1.15.12.10) 20191121
 ------ idealdist_contact.dat (V1.3.4.12) 20191001  IDEAL-DISTANCE CONTACT TERM DATA AS USED IN PROLSQ. VALUES USED HERE ARE BASED ON DATA HARVESTING OF PDB AS OF 20190109.
 ------ restraints for LIG (Unknown) from cif dictionary POB0120.cif
 ------ restraints for CIT (CITRIC ACID) from cif dictionary CIT.cif; generated by GRADE_PDB_LIGAND 1.2.12 (October 4 2016) using MOGUL 1.7.2(RC1), CSD as538be, with quantum mechanics RM1 ((DatabaseID PDB-CIT))
 ------ assume.dat (V1.9.4.2) 20180705
 
 Have picked up spacegroup name for pdb output as 'P 63      '
 Setting up Chain/Residue arrays
 
 Have command line specification -autoncs*
     Have found       1 NCS relations
     Pairwise matching residue number and type between chains: 
                    A        B        S    
          A         .      100%       -   
          B                  .        -   
          S                           .   
       Table key--                                                       
          all   : two chains have 100% match and same number of residues 
         multi  : ditto and they contribute to a multi chain ncs cluster 
          ***%  : two chains have >80% matching residue number/type      
           -    : chains appear to be unrelated                          
      chain A     have identified ncs to     1 other chains. So will weight each autoncs by (       6.666/#)=      6.6660
      chain B     have identified ncs to     1 other chains. So will weight each autoncs by (       6.666/#)=      6.6660
      chain S     have identified ncs to     0 other chains. 
     For .Gelly cards produced see below - after pruning
 
 Setting up BUSTER_SET groups
 Have found    89 BUSTER_SET cards
 Now silently applying automatic/dictionary set definitions. For a verbose listing use '-verbose_set' flag.
 Applying user defined set definitions:
 =====NOTE BUSTER_SET ncsautoXcld = Water --> set 'ncsautoXcld' now has   581 atoms 
 =====NOTE BUSTER_SET ncsautoABset = Chain_A \ ncsautoXcld --> set 'ncsautoABset' now has  2785 atoms 
 Have defined    48 sets: 
      set:                 'All' has   6138 atoms in   1282 residues - automatically defined
      set:               'Empty' has      0 atoms in      0 residues - automatically defined
      set:            'ExoticAA' has      0 atoms in      0 residues - defined by dictionary file
      set:           'ExoticNUC' has      0 atoms in      0 residues - defined by dictionary file
      set:              'NotHET' has   5514 atoms in    698 residues - defined by dictionary file
      set:             'Chain_A' has   2785 atoms in    351 residues - automatically defined
      set:             'Chain_B' has   2772 atoms in    350 residues - automatically defined
      set:             'Chain_S' has    581 atoms in    581 residues - automatically defined
      set:               'Water' has    581 atoms in    581 residues - defined by dictionary file
      set:          'StdProtein' has   5514 atoms in    698 residues - defined by dictionary file
      set:             'Protein' has   5514 atoms in    698 residues - defined by dictionary file
      set:                'Back' has   2794 atoms in    698 residues - defined by dictionary file
      set:                'Side' has   2720 atoms in    650 residues - defined by dictionary file
      set:        'RnaCanonical' has      0 atoms in      0 residues - defined by dictionary file
      set:        'DnaCanonical' has      0 atoms in      0 residues - defined by dictionary file
      set:     'RnaDnaCanonical' has      0 atoms in      0 residues - defined by dictionary file
      set:     'DnaRnaCanonical' has      0 atoms in      0 residues - defined by dictionary file
      set:         'RnaExtended' has      0 atoms in      0 residues - defined by dictionary file
      set:         'DnaExtended' has      0 atoms in      0 residues - defined by dictionary file
      set:      'RnaDnaExtended' has      0 atoms in      0 residues - defined by dictionary file
      set:      'DnaRnaExtended' has      0 atoms in      0 residues - defined by dictionary file
      set:                 'Dna' has      0 atoms in      0 residues - defined by dictionary file
      set:                 'Rna' has      0 atoms in      0 residues - defined by dictionary file
      set:              'RnaDna' has      0 atoms in      0 residues - defined by dictionary file
      set:              'DnaRna' has      0 atoms in      0 residues - defined by dictionary file
      set:             'Nucleic' has      0 atoms in      0 residues - defined by dictionary file
      set:             'Polymer' has   5514 atoms in    698 residues - defined by dictionary file
      set: 'BufferWeichenberger' has     13 atoms in      1 residues - defined by dictionary file
      set:    'BufferAdditional' has      0 atoms in      0 residues - defined by dictionary file
      set:              'Buffer' has     13 atoms in      1 residues - defined by dictionary file
      set:         'MetalAlkali' has      0 atoms in      0 residues - defined by dictionary file
      set:  'MetalAlkalineEarth' has      0 atoms in      0 residues - defined by dictionary file
      set:     'MetalTransition' has      0 atoms in      0 residues - defined by dictionary file
      set: 'MetalPostTransition' has      0 atoms in      0 residues - defined by dictionary file
      set:    'MetalLanthanides' has      0 atoms in      0 residues - defined by dictionary file
      set:               'Metal' has      0 atoms in      0 residues - defined by dictionary file
      set:         'SugarAlphaD' has      0 atoms in      0 residues - defined by dictionary file
      set:         'SugarAlphaL' has      0 atoms in      0 residues - defined by dictionary file
      set:               'Sugar' has      0 atoms in      0 residues - defined by dictionary file
      set:               'Other' has     43 atoms in      3 residues - defined by dictionary file
      set:            'Hydrogen' has      0 atoms in      0 residues - defined by dictionary file
      set:         'nonHydrogen' has   6138 atoms in   1282 residues - defined by dictionary file
      set:             'OccZero' has      0 atoms in      0 residues - defined by dictionary file
      set:          'OccNonZero' has   6138 atoms in   1282 residues - defined by dictionary file
      set:             'OccFull' has   6076 atoms in   1278 residues - defined by dictionary file
      set:          'OccNonFull' has     62 atoms in      4 residues - defined by dictionary file
      set:         'ncsautoXcld' has    581 atoms in    581 residues - user specified
      set:        'ncsautoABset' has   2785 atoms in    351 residues - user specified
 No command line arguments '-occ_allhet' or '-occ_restyp' so will not setup automated occcupancy refinement
 
 Interpreting CONSTANT and FREE cards: 
 =====CONSTANT     OCC
 Have found and interpreted      1 CONSTANT cards in the TNT input. 
 Before any COMBINE - Number of atoms with fixed XYZ =     0
 Before any COMBINE - Number of atoms with fixed B   =     0
 Before any COMBINE - Number of atoms with fixed OCC =  6138
 Before any COMBINE - Number of atoms with fixed BIJ =   581
                            cf total number of atoms =  6138
 
 There are no COMBINE cards to be found in the TNT card input 
 
 There are no NOTE BUSTER_COMBINE_RES cards to be found in the TNT card input 

 ############### Working out full connectivity list


 ############### Working out equivalent atoms


 Residue with most atoms given below:
        A|501:O01(LIG)      
        A|501:C02(LIG)      
        A|501:N03(LIG)      
        A|501:C04(LIG)      
        A|501:C05(LIG)      
        A|501:C06(LIG)      
        A|501:C07(LIG)      
        A|501:C08(LIG)      
        A|501:C09(LIG)      
        A|501:C10(LIG)      
        A|501:C11(LIG)      
        A|501:N12(LIG)      
        A|501:C13(LIG)      
        A|501:C14(LIG)      
        A|501:C15(LIG)      


 Have no '-equiv_check_plus' argument - so will take atom type into
 account when checking for equivalence of otherwise identical atoms.
 

 ############### Looking at dihedrals


 ############### Looking at chirals

 NOTE BUSTER_CHIRAL_ANGLECUT card found - read value=    -31.00 degs from it
 NOTE BUSTER_CHIRAL_SIGMA card found - read value=      1.70 degs from it
 RANGE allowed for B variables in refinement:   from      3.00000 to    300.00000

 ############### Preparing for PDB output


 ############### Processing extra restraints explicitly defined

 No 'NOTE BUSTER_TARGET' cards or -target?? args found (used to read coordinates files for soft ncs to external)
 No 'NOTE BUSTER_OCCSUM'     cards found - so not using sum of occupancy restraints
 No 'NOTE BUSTER_DISTANCE'   cards found - so not setting up any additional atom-atom distance restraints
 No 'NOTE BUSTER_UTILANGLE'  cards found - so not setting up any additional restraints on any angle
 No 'NOTE BUSTER_UTILTOR'    cards found - so not setting up any additional sinusoidal restraints on torsions
 
 --- Have some similarity definitions 
     Similarity restraints on RMSD use superposition routines from: 
     Coutsias, E.A., Seok, C., Dill, K.A.(2004)
     "Using quaternions to calculate RMSD", J. Comput. Chem., 25:1849-1857 

 Have found    1 'NOTE BUSTER_SIM_DEFINE' cards 
     Maximum number of chains involved in any SIM group (excluding the template)=     1


 ########## Interpreting card: NOTE BUSTER_SIM_DEFINE ncsautoAB ncsautoABset B
     Number of atoms in template set=  2785
     Number of related chains (other than template) in SIM set=    1
     List of chains to be restrained (excluding template)=   B   
     Pairwise RMSD, XYZ in Angstroms, for SIM group: ncsautoAB
                 A         B   
     A         -----     ----- 
     B         3.123     ----- 
     Overall RMSD,XYZ for the SIM group is     3.123 Angstroms
     Pairwise rms deviation of [individual B's minus their set/chain average] in Angs**2
                 A         B         Bavg
     A         -----     -----     83.24
     B         27.78     -----     65.76
     Overall RMSD,B   for the SIM group is    27.781 Angs**2
     The number of common atoms for the above fits:
                 A         B   
     A         -----     ----- 
     B          2772     ----- 
     (compared to the template set that has    2785 atoms in it)
     List of atoms that are absent in one or more chains for this group: 
                                    A    B   
                    C1      401     +    -
                    O1      401     +    -
                    O2      401     +    -
                    C2      401     +    -
                    C3      401     +    -
                    O7      401     +    -
                    C4      401     +    -
                    C5      401     +    -
                    O3      401     +    -
                    O4      401     +    -
                    C6      401     +    -
                    O5      401     +    -
                    O6      401     +    -
     (key  +  atom present for this chain 
           -  atom absent  for this chain)
 transforms for each of the superpositions orthogonal matrix r11 r12 r13 r21 r22 r23 r31 r32 r33 tx ty tz (PDBSET syntax): 
TRANS_AB -0.878043 -0.478213 -0.018783 -0.478333  0.878176  0.002195  0.015445  0.010912 -0.999821    34.241    32.806    15.400
 reverse transforms 
TRANS_BA -0.878043 -0.478333  0.015445 -0.478213  0.878176  0.010912 -0.018783  0.002195 -0.999821    45.519   -12.603    15.968
 
 Setting up LSSR interatomic distance restraints
 Interpreting card: NOTE BUSTER_SIM_RESTRAIN_LSSR ncsautoAB 6.666
        setting up interatomic distance pair restraints with:
        Dmin=         0.00
        Dmax=         5.50
        sigma=        0.20
        Vmax=         3.00
        weight=       6.67
        number of distance pairs defined for this set is      35968
 Have not supplied either '-sim_swap_equiv' or '-sim_swap_equiv_plus' argument 
   so will not try swaping equivalent atoms to improve NCS and/or target agreement.
 
 NCS by LSSR outlier analysis
 -autoncs option automatic NCS prune routine -------- 
 Residues pruned out by gradient condition:
          A|253     B|253
  
 Residues pruned out by function/max condition:
          A|149     A|150     A|158     A|167     A|168     A|169     A|170 
          A|171     A|172     A|173     A|174     A|187     A|188     A|215 
          A|216     A|220     A|259     A|264     A|345    A|ENDA     A|501 
          B|149     B|150     B|158     B|167     B|168     B|169     B|170 
          B|171     B|172     B|173     B|174     B|187     B|188     B|215 
          B|216     B|220     B|259     B|264     B|345    B|ENDA     B|501 
       
 Total number of residues for which LSSR restraints have been pruned out is       44 out of      700 participating
 Number of LSSR restraints pruned out is      3006 this is    8.4% of the total           35968
  
 -autoncs .Gelly cards suitable for adaptation for manual definition/pruning
           (note auto pruning is done in a slightly different way so exact results may differ slightly)
 -autoncs option .Gelly cards also written to file  auto_gelly_cards.txt
NOTE BUSTER_SET ncsautoXcld = Water    # card added - produced by -autoncs
NOTE BUSTER_SET ncsautoXcld = ncsautoXcld + { A|149 A|150 A|158 A|167 A|168 }    # card added - produced by -autoncs
NOTE BUSTER_SET ncsautoXcld = ncsautoXcld + { A|169 A|170 A|171 A|172 A|173 }    # card added - produced by -autoncs
NOTE BUSTER_SET ncsautoXcld = ncsautoXcld + { A|174 A|187 A|188 A|215 A|216 }    # card added - produced by -autoncs
NOTE BUSTER_SET ncsautoXcld = ncsautoXcld + { A|220 A|253 A|259 A|264 A|345 }    # card added - produced by -autoncs
NOTE BUSTER_SET ncsautoXcld = ncsautoXcld + { A|ENDA A|501 }    # card added - produced by -autoncs
NOTE BUSTER_SET ncsautoABset = Chain_A \ ncsautoXcld    # card added - produced by -autoncs
NOTE BUSTER_SIM_DEFINE ncsautoAB ncsautoABset B    # card added - produced by -autoncs
NOTE BUSTER_SIM_RESTRAIN_LSSR ncsautoAB 6.666    # card added - produced by -autoncs

 LSSR function contribution before pruning    154268.2
                             after pruning    115736.1
 Graph of which residues pruned from LSSR: ncs_pruned.mtv
 Graph of LSSR function outliers ncs_function_outliers.mtv
 Graph of LSSR gradient outliers ncs_gradient_outliers.mtv
 LSSR - analysis of distance difference distribution for NCS
         DeltaD less than 0.5 Angs:  proportion of population= 96.42% average DeltaD of subset     0.105 Angs
 Histogram of LSSR deltaD distribution ncs_histogram.mtv
 
 Interpreting card: NOTE BUSTER_TLS_SET tls1 { A|* }
 Interpreting card: NOTE BUSTER_TLS_SET tls2 { B|* }
 Interpreting card: NOTE BUSTER_TLS_SET tls1 { A|* }
 Interpreting card: NOTE BUSTER_TLS_T   tls1     -1.04460E-01 -3.29576E-03  1.26753E-01 -2.05452E-02  1.97227E-02  9.29085E-03
 Interpreting card: NOTE BUSTER_TLS_L   tls1      9.04625E-01  1.02472E+00  1.05382E-01  2.72562E-01 -2.87417E-01  3.46645E-01
 Interpreting card: NOTE BUSTER_TLS_S   tls1      2.27101E-01 -2.03443E-01  2.22735E-01 -5.71643E-02 -4.35115E-03 -1.74217E-01 -1.22766E-01 -1.55520E-02
 Interpreting card: NOTE BUSTER_TLS_O   tls1     -2.33111E+01 -8.37601E+01  1.13266E+01
 Interpreting card: NOTE BUSTER_TLS_SET tls2 { B|* }
 Interpreting card: NOTE BUSTER_TLS_T   tls2     -1.36395E-01 -9.35808E-02  2.57140E-01  1.23450E-02  4.34122E-02  4.18694E-03
 Interpreting card: NOTE BUSTER_TLS_L   tls2      6.37772E-01  2.55963E-01  4.61831E-01  3.69121E-01 -3.49623E-01 -2.69037E-01
 Interpreting card: NOTE BUSTER_TLS_S   tls2     -7.13175E-03  7.18486E-02 -5.60545E-02  8.36950E-02  1.18915E-01 -1.98565E-02 -1.56302E-02  2.94633E-02
 Interpreting card: NOTE BUSTER_TLS_O   tls2      2.21121E+01 -7.20987E+01  3.46915E+00
 Interpreting card: NOTE BUSTER_TLS_FIX tls1 ALL
 Interpreting card: NOTE BUSTER_TLS_FIX tls2 ALL


 TLS information. Total number of groups:     2


 Group   Natoms    FixRB   FixTLS   Group Tag    Group Spec
     1     2785      Y        Y     tls1    { A|* }
     2     2772      Y        Y     tls2    { B|* }
 
NOTE BUSTER_TLS_SET tls1 { A|* }
NOTE BUSTER_TLS_T   tls1     -1.04460E-01 -3.29576E-03  1.26753E-01 -2.05452E-02  1.97227E-02  9.29085E-03
NOTE BUSTER_TLS_L   tls1      9.04625E-01  1.02472E+00  1.05382E-01  2.72562E-01 -2.87417E-01  3.46645E-01
NOTE BUSTER_TLS_S   tls1      2.27101E-01 -2.03443E-01  2.22735E-01 -5.71643E-02 -4.35115E-03 -1.74217E-01 -1.22766E-01 -1.55520E-02
NOTE BUSTER_TLS_O   tls1     -2.33111E+01 -8.37601E+01  1.13266E+01
NOTE BUSTER_TLS_FIX tls1 ALL
 
NOTE BUSTER_TLS_SET tls2 { B|* }
NOTE BUSTER_TLS_T   tls2     -1.36395E-01 -9.35808E-02  2.57140E-01  1.23450E-02  4.34122E-02  4.18694E-03
NOTE BUSTER_TLS_L   tls2      6.37772E-01  2.55963E-01  4.61831E-01  3.69121E-01 -3.49623E-01 -2.69037E-01
NOTE BUSTER_TLS_S   tls2     -7.13175E-03  7.18486E-02 -5.60545E-02  8.36950E-02  1.18915E-01 -1.98565E-02 -1.56302E-02  2.94633E-02
NOTE BUSTER_TLS_O   tls2      2.21121E+01 -7.20987E+01  3.46915E+00
NOTE BUSTER_TLS_FIX tls2 ALL


 TLS: mmCIF group specification begin


#
loop_
_pdbx_refine_tls_group.id
_pdbx_refine_tls_group.refine_tls_id
_pdbx_refine_tls_group.beg_PDB_ins_code
_pdbx_refine_tls_group.beg_auth_asym_id
_pdbx_refine_tls_group.beg_auth_seq_id
_pdbx_refine_tls_group.end_PDB_ins_code
_pdbx_refine_tls_group.end_auth_asym_id
_pdbx_refine_tls_group.end_auth_seq_id
_pdbx_refine_tls_group.selection
_pdbx_refine_tls_group.selection_details
   1   1 . A   -1 . A  346 . '{ A|* }'
   2   1 . A  401 . A  501 . '{ A|* }'
   3   2 . B   -1 . B  346 . '{ B|* }'
   4   2 . B  501 . B  501 . '{ B|* }'
#


 TLS: mmCIF group specification end


 Interpreting card: NOTE BUSTER_ANISO_SET Empty
 Interpreting card: NOTE BUSTER_ANISO_WEIGHT SCA  16.0
 Interpreting card: NOTE BUSTER_ANISO_WEIGHT SPH   0.6    0.6
 Interpreting card: NOTE BUSTER_ANISO_WEIGHT SIM   7.8    4.8    2.6    0
 Interpreting card: NOTE BUSTER_ANISO_WEIGHT RIG  21.5   21.5    3.7    0


 After TLS and ANISO: Number of atoms with fixed/combined xyz =     0
 After TLS and ANISO: Number of atoms with fixed/combined B   =     0
 After TLS and ANISO: Number of atoms with fixed/combined OCC =  6138
 After TLS and ANISO: Number of atoms with fixed/combined BIJ =  6138
 After TLS and ANISO: Number of atoms in a TLS group          =  5557
                                     cf total number of atoms =  6138
 

 ############### Setup state vector

 No free_vect_ulim_xyz argument found: using default of  100.000
 No free_vect_llim_xyz argument found: using default of -100.000
 Number of parameters added for TLS description                 =       0
 Number of parameters to describe molecular conformation        =   24552

 ############### Setup free vector

 Number of free parameters to be adjusted in refinement   =   24552

 ############### Setting up own geometry


 No command line argument '-tnt_fn' so will use gelly to do geometry function calculations

 ============= Call TNT functions


 ============= Get TNT symmetry information

 
      Getting symmetry operators from TNT. 
          gelly will classify symmetry using pdb-like convention: 
               SYMOP   SYMMETRY
              NNNMMM   OPERATOR
                1555   X,Y,Z
                2555   -Y,X-Y,Z
                3555   -X+Y,-X,Z
                4555   -X,-Y,1/2+Z
                5555   Y,-X+Y,1/2+Z
                6555   X-Y,X,1/2+Z
          where NNN -> operator number and MMM -> translation vector
 

 ============= Own Gelly geometry/restraints setup


 ----------- Identify bond-types


    Setting up GEOMBONDREC_TYPE_HASH* to be able to map a given bond type (e.g.
    "single") to an internal integer (GBOND_TYPE=1) etc.

    Using default  GEOMBONDREC_TYPE_HASH_STR='SING* 1 DOUB* 2 TRIP* 3 AROM* 4 DELO* 4 METAL* 5':
    to override default use a NOTE BUSTER_GEOMBONDREC_TYPE_HASH_STR gelly card.

 ----------- Classify torsion into peptide omega and other


 ----------- Setup of non-bonded terms

 Ideal-distance contact term setup:
    Restraint function constants (normally these values are from NOTE BUSTER_IDEAL_CONTACT_* cards)
    Sigma for general contacts      0.200 Angs
    Sigma for 1-4 contacts          0.100 Angs (contacts across a torsion)
    Sigma for 1-5 contacts          0.150 Angs
    Sigma for 1-N contacts          0.200 Angs
    Sigma for HBOND pairs           0.100 Angs (contacts between HB_types A and D or B's)
    Contact distance increment for each atom in HBOND          -0.150 Angs 
    Contact distance increment for H-bond acceptor              0.100 Angs 
    Contact distance increment for 1-4 with an O atom          -0.050 Angs 
    Contact distance increment for 1-4 with an N atom          -0.180 Angs 
    Contact distance increment for 1-4 with an C atom          -0.370 Angs 
    Contact distance increment for 1-4 with an S atom          -0.150 Angs 
    Contact distance increment for 1-4 with an P atom          -0.150 Angs 
    Contact distance increment for 1-4 with any other atom     -0.150 Angs 
    All atoms matched for CCP4 atom type.
    Number of CCP4 atom types used is         23 (H-bound and not H-bound counted separately)

 Setting up short/ideal-distance contacts
   Using gelly special position handling. (For TNT instead rerun with argument '-special_tnt')

   No command line argument '-special_all' specified. We will auto-generate
   EXCLUDE cards only for atom-atom contacts across symmetry (i.e. on special
   position) when this involves a contact within a residue to itself.

   Have set up non-bonded exclusions - 1-3 contacts
   Atom A|27:CA         makes the most 1-3 contacts:  6

   Have set up non-bonded exclusions - 1-4 contacts
   Atom A|27:N          makes the most 1-4 contacts:  7

   Have set up non-bonded exclusions - 1-5 contacts
   Atom A|28:C.A        makes the most 1-5 contacts: 10

 ############################################################################################################
 ###################################################### setting up contact terms
 ############################################################################################################


 ============= Call Gelly versions of TNT functions

 WARNING: 1-4 contact without 1-(2,3)-4 data for A|401:O2(CIT)        and A|401:C3(CIT)        X-X
 WARNING: 1-4 contact without 1-(2,3)-4 data for A|401:C2(CIT)        and A|401:O6(CIT)        X-X
 WARNING: 1-4 contact without 1-(2,3)-4 data for A|501:C02(LIG)       and A|501:C05(LIG)       X-X
 WARNING: 1-4 contact without 1-(2,3)-4 data for A|501:C04(LIG)       and A|501:C11(LIG)       X-X
 WARNING: 1-4 contact without 1-(2,3)-4 data for B|501:C02(LIG)       and B|501:C13(LIG)       X-X
 WARNING: 1-4 contact without 1-(2,3)-4 data for B|501:C04(LIG)       and B|501:C11(LIG)       X-X

       TERM      weight      number      TNT_fn    Gelly_fn        diff
       BOND       2.000        5676    8751.558    8751.558       0.000
       ANGL       2.000        7707   16684.232   16684.232      -0.000
       IMPR       0.000           0       0.000       0.000       0.000
       TORS       2.000        1979    3207.584    3207.584      -0.000
       TRIG       2.000           0       0.000       0.000       0.000
       PLAN       5.000         966   16908.656   16908.656       0.000
       CONT       5.000           0    6684.955       0.000    6684.955
       BCOR      10.000        5630  194009.907  198436.830   -4426.924


 ============= Call Gelly geometry functions

   Switching torsion functional form to sinusoidal rather than split harmonic functional.
      if you want this not to happen then rerun with '-torsharm'
      Weight for sinusoidal torsion=     2.000
   BCOR function/gradient will be split between H and non-H atoms (as for any atom pair). Use '-bcorrel_all_in_hydrogens' to change this.

 ----------- Fix B-correlation restraints

 
 Checking for bonds that do not have a bcorrel restraint defined
 We will wipe all existing BCORrels if OCC=0.0 atom(s) is/are involved. This can be' switched off via the '-bcorrel_nowipe_zeroocc' flag.
 We will wipe all existing BCORrels if only non-hydrogen atom(s) with fixed B(iso) are involved. This can be switched off via the '-bcorrel_nowipe_fixb_nonhydrogens' flag.
 We will keep all existing BCORrels if hydrogen atom(s) with fixed B(iso) is/are involved. This can be switched off via the '-bcorrel_wipe_fixb_hydrogens' flag.
 No -bcorrel_missing_sigma found. So will set sigma to     2.5 Ang**2 for any bond with no existing BCORrel
 No -bcorrel_missing_sigma_hydrogens found. So will set sigma to     1.0 Ang**2 for any bond with no existing BCORrel involving hydrogens

 No active BCORels found involving OCC=0.0 atom(s)


 No active BCORels found involving fixed-B (only) non-hydrogen atom(s)

    add a default BCOR for bond A|401:C1(CIT) A|401:C2(CIT)
    add a default BCOR for bond A|401:C1(CIT) A|401:O1(CIT)
    add a default BCOR for bond A|401:C1(CIT) A|401:O2(CIT)
    add a default BCOR for bond A|401:C2(CIT) A|401:C3(CIT)
    add a default BCOR for bond A|401:C3(CIT) A|401:C4(CIT)
    add a default BCOR for bond A|401:C3(CIT) A|401:C6(CIT)
    add a default BCOR for bond A|401:C3(CIT) A|401:O7(CIT)
    add a default BCOR for bond A|401:C4(CIT) A|401:C5(CIT)
    add a default BCOR for bond A|401:C5(CIT) A|401:O3(CIT)
    add a default BCOR for bond A|401:C5(CIT) A|401:O4(CIT)
    add a default BCOR for bond A|401:C6(CIT) A|401:O5(CIT)
    add a default BCOR for bond A|401:C6(CIT) A|401:O6(CIT)
    add a default BCOR for bond A|501:O01(LIG) A|501:C02(LIG)
    add a default BCOR for bond A|501:C02(LIG) A|501:N03(LIG)
    add a default BCOR for bond A|501:C02(LIG) A|501:C11(LIG)
    add a default BCOR for bond A|501:N03(LIG) A|501:C04(LIG)
    add a default BCOR for bond A|501:C04(LIG) A|501:C05(LIG)
    add a default BCOR for bond A|501:C04(LIG) A|501:C09(LIG)
    add a default BCOR for bond A|501:C05(LIG) A|501:C06(LIG)
    add a default BCOR for bond A|501:C05(LIG) A|501:C10(LIG)
    add a default BCOR for bond A|501:C06(LIG) A|501:C07(LIG)
    add a default BCOR for bond A|501:C07(LIG) A|501:C08(LIG)
    add a default BCOR for bond A|501:C08(LIG) A|501:C09(LIG)
    add a default BCOR for bond A|501:C10(LIG) A|501:C11(LIG)
    add a default BCOR for bond A|501:C11(LIG) A|501:N12(LIG)
    add a default BCOR for bond A|501:C11(LIG) A|501:C15(LIG)
    add a default BCOR for bond A|501:N12(LIG) A|501:C13(LIG)
    add a default BCOR for bond A|501:C13(LIG) A|501:C14(LIG)
    add a default BCOR for bond A|501:C14(LIG) A|501:C15(LIG)
    add a default BCOR for bond B|501:O01(LIG) B|501:C02(LIG)
    add a default BCOR for bond B|501:C02(LIG) B|501:N03(LIG)
    add a default BCOR for bond B|501:C02(LIG) B|501:C11(LIG)
    add a default BCOR for bond B|501:N03(LIG) B|501:C04(LIG)
    add a default BCOR for bond B|501:C04(LIG) B|501:C05(LIG)
    add a default BCOR for bond B|501:C04(LIG) B|501:C09(LIG)
    add a default BCOR for bond B|501:C05(LIG) B|501:C06(LIG)
    add a default BCOR for bond B|501:C05(LIG) B|501:C10(LIG)
    add a default BCOR for bond B|501:C06(LIG) B|501:C07(LIG)
    add a default BCOR for bond B|501:C07(LIG) B|501:C08(LIG)
    add a default BCOR for bond B|501:C08(LIG) B|501:C09(LIG)
    add a default BCOR for bond B|501:C10(LIG) B|501:C11(LIG)
    add a default BCOR for bond B|501:C11(LIG) B|501:N12(LIG)
    add a default BCOR for bond B|501:C11(LIG) B|501:C15(LIG)
    add a default BCOR for bond B|501:N12(LIG) B|501:C13(LIG)
    add a default BCOR for bond B|501:C13(LIG) B|501:C14(LIG)
    add a default BCOR for bond B|501:C14(LIG) B|501:C15(LIG)

 Have added a total of        46 BCORrels for bonds which do not already have a BCORrel


 No bonds (for potential BCORrel addition) were skipped due to both atoms having constant/fixed B(iso)


 ----------- Deactivate hydrogen-free planes if hydrogen atoms present

 A total of       0 planes have been deactivated because they are duplicated hydrogen-free and with-hydrogen forms.

 ----------- Handle peptide omega angle(s)

      Torsion angles split into peptide omega and other
         Number of peptide omega angles is       698
         Number of other torsion is             1281
      Peptide omega angles are monitored rather than directly restrained (PLANE used instead).

 ----------- Fix cis-PRO

 Now apply the fix to get correct restraints for cis PRO residues
   Have got      11 bonds records and      26 angles
   from GEOMETRY cards for the special PROC residue
   Weight for ideal-distance contact term=     4.000

 ----------- Prepare SSBOND/LINK for output PDB file


 ----------- Prepare for QM


 ----------- Prepare symmetry in utility distance restraints


 ############### Setting up Rhofit contacts (if needed)

 No ligand-(fixed protein) contact term will be used

 ############### Working out initial function and gradient


   MaxLik Scaling Cycle 0002
 
 Initial functional value = 5.822281E+05 rms gradient=      898.626
 X-ray contribution to function value = 1.851183E+05
 Geometry contribution (inc ncs)      = 3.971098E+05
 Geometry function summary
     term                           number     weight       rms        GooF      function     fn/numb
     ================================================================================================
     BOND bond lengths (angs)       5676       2.000      0.0176       0.878      8751.56       1.542
     ANGL bond angles (degs)        7707       2.000        1.75       1.040     16684.23       2.165
     TORS split-harmonic tors       1281       0.000       17.84       1.119         0.00       0.000
     SINTOR sinusoidal tors         1281       2.000       17.84                  2439.67       1.905
     PLAN general plane (angs)       966       5.000      0.0374       1.871     16908.66      17.504
     BCORrelations (angs^2)         5676      10.000       5.299       1.892    203226.55      35.805
     CONT (bad contacts)               0       5.000                                 0.00
     IDEAL(ideal-dist contact)      5446       4.000                             33326.08
     CHIRAL (gelly semiharmon)       740       5.000                                36.93
     SIM similarity restraint for NCS and/or TARGET                             115736.11
     OCCSUM restraint on sum of occupanies                                           0.00       as none defined
     DISTAN utility restraints interatomic distance                                  0.00       as none defined
     UTILANGLE utility angle restraints                                              0.00       as none defined
     UTILTOR utility restraints on torsion angles                                    0.00       as none defined
     Peptide omega torsion angles                           7.93 degs - monitor only
 
 WARNING: Have |delta/sigma| deviations > 5.0 sigma. Number of outliers for each term: 
 WARNING:         5 bond lengths. Worst is    20.8 sigs     1.96 Angs A|501:C10=C05 (LIG)
 WARNING:        11 bond angles.  Worst is    27.2 sigs    38.39 degs A|501:C10=C05=C04 (LIG)
 WARNING:        14 planes.       Worst is     6.4 sigs     0.13 Angs A|401:O7=C3=C6=O6 (CIT)
 WARNING:        20 idealD contacts. Worst    14.5 sigs     2.08 Angs A|174:N(THR)=A|175:N(GLN)
 WARNING:       111 Bcorrel.      Worst is    13.8 sigs    34.57 Angs^2 B|344:C(LYS)=B|345:N(LEU)

 Weighted rms      0.0176         1.748  (temporary rms bond and angle for BUSTER to pick up!)
 Weighted intra-residual rms(bond) for StdProtein (excluding H)      0.0150
 Weighted intra-residual rms(bond) for Buffer     (excluding H)      0.0272

 ############### Running initial check


 Initial Geometry Sanity Check

 Check for duplicate bonds: no duplicates found
 
 Check for duplicate or missing bond angles: all are OK
 Check for bond between an atom and itself not involving symmetry:  all are OK
 *** WARNING: There are 13 bad contacts where Delta/sigma is greater than      6.5
 *** WARNING: Looking at each one in turn to see whether it is likely to indicate a setup problem with geometry restraints.

        1 dist=   2.08 idealD=   2.80 sigma=   0.05 abs(Delta/sigma)=   14.51
          Between atoms   A|174:N (THR)           and   A|175:N (GLN)           (N-N)
          This is a contact between two 'Protein' type atoms from distinct
          residues.
 [check-042]
          Not between atoms that normally join residues in proteins. So
          probably just a bad contact (maybe due to a poor or very distorted
          input model).

        2 dist=   1.02 idealD=   3.84 sigma=   0.22 abs(Delta/sigma)=   12.98
          Between atoms   A|169:CA (GLY)          and   A|501:C13 (LIG)         (C-C)
          occupancies=     1.00        0.44
 [check-049]
 WARNING: Could be a bad contact but may well be an unrecognized link from
          protein to HETATM residue? It is possible restraints have not been
          properly set up.
          If you want BUSTER to regard this as a bond try adding the
          following card before the CRYST1 line in the input pdb file:
LINK         CA  GLY A 169                 C13 LIG A 501     1555   1555  1.02  

        3 dist=   1.30 idealD=   3.47 sigma=   0.22 abs(Delta/sigma)=    9.70
          Between atoms   A|173:CE (LYS)          and   A|175:O (GLN)           (C-O)
          This is a contact between two 'Protein' type atoms from distinct
          residues.
 [check-042]
          Not between atoms that normally join residues in proteins. So
          probably just a bad contact (maybe due to a poor or very distorted
          input model).

        4 dist=   2.26 idealD=   3.85 sigma=   0.17 abs(Delta/sigma)=    9.64
          Between atoms   A|169:C (GLY)           and   A|501:N12 (LIG)         (C-N)
          occupancies=     1.00        0.44
 [check-049]
          Could be a bad contact but may well be an unrecognized link from
          protein to HETATM residue? It is possible restraints have not been
          properly set up.

        5 dist=   2.39 idealD=   3.45 sigma=   0.12 abs(Delta/sigma)=    9.24
          Between atoms   A|284:O (GLN)           and   A|501:C04 (LIG)         (O-C)
          occupancies=     1.00        0.44
          The contact is between two atoms via a symmetry operator.
 [check-012]
          One atom has occupancy below 1. Hopefully refinement will fix this.

        6 dist=   1.94 idealD=   3.51 sigma=   0.18 abs(Delta/sigma)=    8.50
          Between atoms   A|284:NE2 (GLN)         and   A|501:C08 (LIG)         (N-C)
          occupancies=     1.00        0.44
          The contact is between two atoms via a symmetry operator.
 [check-012]
          One atom has occupancy below 1. Hopefully refinement will fix this.

        7 dist=   2.14 idealD=   3.73 sigma=   0.19 abs(Delta/sigma)=    8.47
          Between atoms   A|169:N (GLY)           and   A|501:C13 (LIG)         (N-C)
          occupancies=     1.00        0.44
 [check-049]
          Could be a bad contact but may well be an unrecognized link from
          protein to HETATM residue? It is possible restraints have not been
          properly set up.

        8 dist=   1.95 idealD=   3.66 sigma=   0.20 abs(Delta/sigma)=    8.46
          Between atoms   A|169:C (GLY)           and   A|501:C13 (LIG)         (C-C)
          occupancies=     1.00        0.44
 [check-049]
 WARNING: Could be a bad contact but may well be an unrecognized link from
          protein to HETATM residue? It is possible restraints have not been
          properly set up.
          If you want BUSTER to regard this as a bond try adding the
          following card before the CRYST1 line in the input pdb file:
LINK         C   GLY A 169                 C13 LIG A 501     1555   1555  1.95  

        9 dist=   2.17 idealD=   3.73 sigma=   0.19 abs(Delta/sigma)=    8.33
          Between atoms   A|169:CA (GLY)          and   A|501:N12 (LIG)         (C-N)
          occupancies=     1.00        0.44
 [check-049]
          Could be a bad contact but may well be an unrecognized link from
          protein to HETATM residue? It is possible restraints have not been
          properly set up.

       10 dist=   2.05 idealD=   3.51 sigma=   0.18 abs(Delta/sigma)=    7.91
          Between atoms   A|284:NE2 (GLN)         and   A|501:C09 (LIG)         (N-C)
          occupancies=     1.00        0.44
          The contact is between two atoms via a symmetry operator.
 [check-012]
          One atom has occupancy below 1. Hopefully refinement will fix this.

       11 dist=   1.73 idealD=   3.47 sigma=   0.22 abs(Delta/sigma)=    7.77
          Between atoms   A|173:CD (LYS)          and   A|175:O (GLN)           (C-O)
          This is a contact between two 'Protein' type atoms from distinct
          residues.
 [check-042]
          Not between atoms that normally join residues in proteins. So
          probably just a bad contact (maybe due to a poor or very distorted
          input model).

       12 dist=   2.28 idealD=   3.73 sigma=   0.19 abs(Delta/sigma)=    7.72
          Between atoms   A|170:N (GLU)           and   A|501:C13 (LIG)         (N-C)
          occupancies=     1.00        0.44
 [check-049]
          Could be a bad contact but may well be an unrecognized link from
          protein to HETATM residue? It is possible restraints have not been
          properly set up.

       13 dist=   2.34 idealD=   3.84 sigma=   0.22 abs(Delta/sigma)=    6.92
          Between atoms   A|169:CA (GLY)          and   A|501:C14 (LIG)         (C-C)
          occupancies=     1.00        0.44
 [check-049]
          Could be a bad contact but may well be an unrecognized link from
          protein to HETATM residue? It is possible restraints have not been
          properly set up.
 GEOMETRY CHECK OVERALL:  WARN


 ############### Creating geometry/screen report


 ############### Option "tao" - run Tao optimizer

 TAO: Toolkit for Advanced Optimization. Reference: 
     Munson, T., Sarich, J., Wild, S., Benson, S., McInnes, L.C. (2018)
     "TAO User Manual (Revision 3.9)", Mathematics and Computer Science
     Division, Argonne National Laboratory
     ANL/MCS-TM-322, http://www.mcs.anl.gov/petsc/
 
 Have found -keeppdb flag so will write out a .pdb file every 1000000 its during opt
 Will apply TAO optimization 
 Maximum number of iterations      100
 Convergence test on Grms       4.0000 (-glim)
 Limit on rms displacement to original position (rmsD2init): NONE test inactive (-dlim)

    analysing argument no 0 : -autoncs with j=0
    analysing argument no 1 : -hydrogens_input_occ2zero with j=0
    analysing argument no 2 : -hydrogens_output_occ2heavy with j=0
    analysing argument no 3 : -screen with j=0
    analysing argument no 4 : 100 with j=0
    analysing argument no 5 : -screen_sigma with j=0
    analysing argument no 6 : 3.0 with j=0
    analysing argument no 7 : -keeppdb with j=0
    analysing argument no 8 : 1000000 with j=0
    analysing argument no 9 : -taomonitor_verbose with j=0
    found argument starting with "-tao" : -taomonitor_verbose
    analysing argument no 10 : 2 with j=1
    found value after -tao/-options_/-viewer_ argument : "2"
 PETSC/TAO: setting option "-taomonitor_verbose" = "2"


   MaxLik Scaling Cycle 0003
   Iters     Tot_Funct    Xray_Funct    Geom_Funct          Grms     rmsD2init     maxD2init             R         Rfree           LLG       LLGfree     rms(bond)    rms(angle)       Opttag_H
   Iters             K   B_IMPF_FRAG        K_MISS   B_TEMP_MISS   K_IMPF_MISS   B_IMPF_MISS        K_SOLV   B_TEMP_SOLV   K_IMPF_SOLV   B_IMPF_SOLV        B_TEMP     B_TEMP_11     B_TEMP_12     B_TEMP_22     B_TEMP_13     B_TEMP_23     B_TEMP_33      K_WILSON      B_WILSON      FGCYCLES       Scatag_H
   Iters          BOND         ANGLE      IMPROPER       TORSION    SINTORSION      TRIGONAL        PLANAR  BCORRELATION       CONTACT     IDEALDIST        CHIRAL    SIMILARITY       OCCSSUM  UTILDISTANCE     UTILANGLE   UTILTORSION        RHOFIT        ADP-01        ADP-02        ADP-03        ADP-04        ADP-05        ADP-06        ADP-07        ADP-08        ADP-09        ADP-10      RHOFITPC            QM       Funtag_H
       0  5.822281E+05  1.851183E+05  3.971098E+05       898.626  0.000000E+00  0.000000E+00      0.213534      0.217737  0.000000E+00  0.000000E+00     0.0176184       1.74842       Opttag_D
       0       45.0296       2.28555       1.00000  0.000000E+00       1.00000  0.000000E+00      0.787002       73.3785       1.00000       24.6496  0.000000E+00       2.33098  0.000000E+00       2.33098  0.000000E+00  0.000000E+00      -4.66196       44.9909       1.47392        2       Scatag_D
       0       8751.56       16684.2  0.000000E+00  0.000000E+00       2439.67  0.000000E+00       16908.7  2.032265E+05  0.000000E+00       33326.1       36.9279  1.157361E+05 -1.000000E+10 -1.000000E+10 -1.000000E+10 -1.000000E+10 -1.000000E+10  0.000000E+00  0.000000E+00  0.000000E+00  0.000000E+00  0.000000E+00  0.000000E+00  0.000000E+00  0.000000E+00  0.000000E+00  0.000000E+00  0.000000E+00  0.000000E+00       Funtag_D
   Geometry Restraint Deviations SCREEN Information

 

   MaxLik Scaling Cycle 0004

   MaxLik Scaling Cycle 0005

   Wilson plot

   Statistics in resolution bins after refinement cycle 0001
```


|  |  |
| --- | --- |
|  | - Rxpct factors plot- Rxpct factors table- <Log-Lik Gain>- Ln Sigma A- Correlation Coefficients |


```
   Iters     Tot_Funct    Xray_Funct    Geom_Funct          Grms     rmsD2init     maxD2init             R         Rfree           LLG       LLGfree     rms(bond)    rms(angle)
   Iters             K   B_IMPF_FRAG        K_MISS   B_TEMP_MISS   K_IMPF_MISS   B_IMPF_MISS        K_SOLV   B_TEMP_SOLV   K_IMPF_SOLV   B_IMPF_SOLV        B_TEMP     B_TEMP_11     B_TEMP_12     B_TEMP_22     B_TEMP_13     B_TEMP_23     B_TEMP_33      K_WILSON      B_WILSON      FGCYCLES
   Iters          BOND         ANGLE      IMPROPER       TORSION    SINTORSION      TRIGONAL        PLANAR  BCORRELATION       CONTACT     IDEALDIST        CHIRAL    SIMILARITY       OCCSSUM  UTILDISTANCE     UTILANGLE   UTILTORSION        RHOFIT        ADP-01        ADP-02        ADP-03        ADP-04        ADP-05        ADP-06        ADP-07        ADP-08        ADP-09        ADP-10      RHOFITPC            QM
       1  4.626438E+05  1.860399E+05  2.766038E+05       543.195    0.00680591     0.0898515      0.214910      0.219046   -0.00557770   -0.00544559     0.0145847       1.63295       Opttag_D
       1       44.9159       2.33447       1.00000  0.000000E+00       1.00000  0.000000E+00      0.788077       73.0786       1.00000       24.2448  0.000000E+00       2.59076  0.000000E+00       2.59076  0.000000E+00  0.000000E+00      -5.18152       44.9909       1.47392        2       Scatag_D
       1       5997.17       14553.3  0.000000E+00  0.000000E+00       2428.47  0.000000E+00       14929.6  1.001979E+05  0.000000E+00       31074.4       26.3694  1.073966E+05 -1.000000E+10 -1.000000E+10 -1.000000E+10 -1.000000E+10 -1.000000E+10  0.000000E+00  0.000000E+00  0.000000E+00  0.000000E+00  0.000000E+00  0.000000E+00  0.000000E+00  0.000000E+00  0.000000E+00  0.000000E+00  0.000000E+00  0.000000E+00       Funtag_D
   Geometry Restraint Deviations SCREEN Information

 

   MaxLik Scaling Cycle 0006

   Wilson plot

   Statistics in resolution bins after refinement cycle 0002
```


|  |  |
| --- | --- |
|  | - Rxpct factors plot- Rxpct factors table- <Log-Lik Gain>- Ln Sigma A- Correlation Coefficients |


```
   Iters     Tot_Funct    Xray_Funct    Geom_Funct          Grms     rmsD2init     maxD2init             R         Rfree           LLG       LLGfree     rms(bond)    rms(angle)
   Iters             K   B_IMPF_FRAG        K_MISS   B_TEMP_MISS   K_IMPF_MISS   B_IMPF_MISS        K_SOLV   B_TEMP_SOLV   K_IMPF_SOLV   B_IMPF_SOLV        B_TEMP     B_TEMP_11     B_TEMP_12     B_TEMP_22     B_TEMP_13     B_TEMP_23     B_TEMP_33      K_WILSON      B_WILSON      FGCYCLES
   Iters          BOND         ANGLE      IMPROPER       TORSION    SINTORSION      TRIGONAL        PLANAR  BCORRELATION       CONTACT     IDEALDIST        CHIRAL    SIMILARITY       OCCSSUM  UTILDISTANCE     UTILANGLE   UTILTORSION        RHOFIT        ADP-01        ADP-02        ADP-03        ADP-04        ADP-05        ADP-06        ADP-07        ADP-08        ADP-09        ADP-10      RHOFITPC            QM
       2  4.172089E+05  1.863562E+05  2.308526E+05       377.471     0.0118906     0.0925306      0.215542      0.219775   -0.00749167   -0.00773262     0.0127599       1.55774       Opttag_D
       2       44.8544       2.34752       1.00000  0.000000E+00       1.00000  0.000000E+00      0.788735       72.7060       1.00000       24.1654  0.000000E+00       2.70970  0.000000E+00       2.70970  0.000000E+00  0.000000E+00      -5.41940       44.9909       1.47392        1       Scatag_D
       2       4590.35       13243.6  0.000000E+00  0.000000E+00       2418.36  0.000000E+00       13406.5       67859.8  0.000000E+00       29405.4       28.1625       99900.5 -1.000000E+10 -1.000000E+10 -1.000000E+10 -1.000000E+10 -1.000000E+10  0.000000E+00  0.000000E+00  0.000000E+00  0.000000E+00  0.000000E+00  0.000000E+00  0.000000E+00  0.000000E+00  0.000000E+00  0.000000E+00  0.000000E+00  0.000000E+00       Funtag_D
   Geometry Restraint Deviations SCREEN Information

 
       3  3.789056E+05  1.870688E+05  1.918368E+05       331.825     0.0226345      0.179899      0.216908      0.221538    -0.0118038    -0.0127389     0.0129585       1.48998       Opttag_D
       3       44.7844       2.39058       1.00000  0.000000E+00       1.00000  0.000000E+00      0.790213       72.0782       1.00000       24.0339  0.000000E+00       2.90159  0.000000E+00       2.90159  0.000000E+00  0.000000E+00      -5.80317       44.9909       1.47392        1       Scatag_D
       3       4734.40       12116.6  0.000000E+00  0.000000E+00       2402.24  0.000000E+00       10879.5       49328.9  0.000000E+00       26688.4       65.6275       85621.1 -1.000000E+10 -1.000000E+10 -1.000000E+10 -1.000000E+10 -1.000000E+10  0.000000E+00  0.000000E+00  0.000000E+00  0.000000E+00  0.000000E+00  0.000000E+00  0.000000E+00  0.000000E+00  0.000000E+00  0.000000E+00  0.000000E+00  0.000000E+00       Funtag_D
       4  3.577380E+05  1.875722E+05  1.701658E+05       331.740     0.0316242      0.254115      0.217909      0.222872    -0.0148497    -0.0162157     0.0137941       1.58469       Opttag_D
       4       44.7296       2.41832       1.00000  0.000000E+00       1.00000  0.000000E+00      0.791117       71.5768       1.00000       24.0072  0.000000E+00       3.01695  0.000000E+00       3.01695  0.000000E+00  0.000000E+00      -6.03391       44.9909       1.47392        1       Scatag_D
       4       5364.61       13705.8  0.000000E+00  0.000000E+00       2392.79  0.000000E+00       9259.71       40292.2  0.000000E+00       24944.7       109.952       74096.0 -1.000000E+10 -1.000000E+10 -1.000000E+10 -1.000000E+10 -1.000000E+10  0.000000E+00  0.000000E+00  0.000000E+00  0.000000E+00  0.000000E+00  0.000000E+00  0.000000E+00  0.000000E+00  0.000000E+00  0.000000E+00  0.000000E+00  0.000000E+00       Funtag_D
       5  3.405318E+05  1.877784E+05  1.527534E+05       245.560     0.0380782      0.304471      0.218436      0.223680    -0.0160975    -0.0177333     0.0132019       1.50417       Opttag_D
       5       44.6796       2.42133       1.00000  0.000000E+00       1.00000  0.000000E+00      0.791448       71.1548       1.00000       24.0837  0.000000E+00       3.07618  0.000000E+00       3.07618  0.000000E+00  0.000000E+00      -6.15236       44.9909       1.47392        1       Scatag_D
       5       4913.87       12348.3  0.000000E+00  0.000000E+00       2387.53  0.000000E+00       8388.71       34251.5  0.000000E+00       23933.2       125.425       66404.9 -1.000000E+10 -1.000000E+10 -1.000000E+10 -1.000000E+10 -1.000000E+10  0.000000E+00  0.000000E+00  0.000000E+00  0.000000E+00  0.000000E+00  0.000000E+00  0.000000E+00  0.000000E+00  0.000000E+00  0.000000E+00  0.000000E+00  0.000000E+00       Funtag_D
       6  3.202303E+05  1.883745E+05  1.318558E+05       304.399     0.0535762      0.412230      0.219857      0.225523    -0.0197051    -0.0218124     0.0124932       1.62860       Opttag_D
       6       44.5782       2.43962       1.00000  0.000000E+00       1.00000  0.000000E+00      0.792119       70.2366       1.00000       24.2972  0.000000E+00       3.20180  0.000000E+00       3.20180  0.000000E+00  0.000000E+00      -6.40361       44.9909       1.47392        1       Scatag_D
       6       4400.46       14475.9  0.000000E+00  0.000000E+00       2381.32  0.000000E+00       6980.51       28425.4  0.000000E+00       22100.8       158.836       52932.5 -1.000000E+10 -1.000000E+10 -1.000000E+10 -1.000000E+10 -1.000000E+10  0.000000E+00  0.000000E+00  0.000000E+00  0.000000E+00  0.000000E+00  0.000000E+00  0.000000E+00  0.000000E+00  0.000000E+00  0.000000E+00  0.000000E+00  0.000000E+00       Funtag_D
       7  3.100924E+05  1.887373E+05  1.213551E+05       290.985     0.0619402      0.459024      0.220685      0.226580    -0.0219001    -0.0243194     0.0119587       1.64316       Opttag_D
       7       44.5202       2.45239       1.00000  0.000000E+00       1.00000  0.000000E+00      0.792359       69.7756       1.00000       24.4078  0.000000E+00       3.26577  0.000000E+00       3.26577  0.000000E+00  0.000000E+00      -6.53153       44.9909       1.47392        1       Scatag_D
       7       4031.97       14735.8  0.000000E+00  0.000000E+00       2381.16  0.000000E+00       6502.81       24469.9  0.000000E+00       21264.8       142.357       47826.3 -1.000000E+10 -1.000000E+10 -1.000000E+10 -1.000000E+10 -1.000000E+10  0.000000E+00  0.000000E+00  0.000000E+00  0.000000E+00  0.000000E+00  0.000000E+00  0.000000E+00  0.000000E+00  0.000000E+00  0.000000E+00  0.000000E+00  0.000000E+00       Funtag_D
       8  3.028501E+05  1.886510E+05  1.141991E+05       173.167     0.0624087      0.454271      0.220581      0.226541    -0.0213780    -0.0238203     0.0109818       1.52802       Opttag_D
       8       44.5007       2.44116       1.00000  0.000000E+00       1.00000  0.000000E+00      0.792207       69.7142       1.00000       24.4231  0.000000E+00       3.26558  0.000000E+00       3.26558  0.000000E+00  0.000000E+00      -6.53117       44.9909       1.47392        1       Scatag_D
       8       3400.19       12743.1  0.000000E+00  0.000000E+00       2378.97  0.000000E+00       6488.63       21339.0  0.000000E+00       21145.6       107.220       46596.5 -1.000000E+10 -1.000000E+10 -1.000000E+10 -1.000000E+10 -1.000000E+10  0.000000E+00  0.000000E+00  0.000000E+00  0.000000E+00  0.000000E+00  0.000000E+00  0.000000E+00  0.000000E+00  0.000000E+00  0.000000E+00  0.000000E+00  0.000000E+00       Funtag_D
       9  2.947541E+05  1.887039E+05  1.060501E+05       137.446     0.0667536      0.460995      0.220813      0.226996    -0.0216986    -0.0242300     0.0100839       1.48403       Opttag_D
       9       44.4535       2.43460       1.00000  0.000000E+00       1.00000  0.000000E+00      0.792170       69.4409       1.00000       24.4654  0.000000E+00       3.28905  0.000000E+00       3.28905  0.000000E+00  0.000000E+00      -6.57810       44.9909       1.47392        1       Scatag_D
       9       2866.91       12019.9  0.000000E+00  0.000000E+00       2375.58  0.000000E+00       6278.91       18833.4  0.000000E+00       20610.9       70.1247       42994.4 -1.000000E+10 -1.000000E+10 -1.000000E+10 -1.000000E+10 -1.000000E+10  0.000000E+00  0.000000E+00  0.000000E+00  0.000000E+00  0.000000E+00  0.000000E+00  0.000000E+00  0.000000E+00  0.000000E+00  0.000000E+00  0.000000E+00  0.000000E+00       Funtag_D
      10  2.895421E+05  1.888531E+05  1.006890E+05       160.999     0.0720879      0.472342      0.221226      0.227629    -0.0226010    -0.0252324    0.00988806       1.49400       Opttag_D
      10       44.4120       2.43655       1.00000  0.000000E+00       1.00000  0.000000E+00      0.792284       69.1165       1.00000       24.5197  0.000000E+00       3.31578  0.000000E+00       3.31578  0.000000E+00  0.000000E+00      -6.63155       44.9909       1.47392        1       Scatag_D
      10       2756.61       12181.9  0.000000E+00  0.000000E+00       2373.63  0.000000E+00       6006.72       17363.8  0.000000E+00       20022.9       61.3028       39922.2 -1.000000E+10 -1.000000E+10 -1.000000E+10 -1.000000E+10 -1.000000E+10  0.000000E+00  0.000000E+00  0.000000E+00  0.000000E+00  0.000000E+00  0.000000E+00  0.000000E+00  0.000000E+00  0.000000E+00  0.000000E+00  0.000000E+00  0.000000E+00       Funtag_D
      11  2.834371E+05  1.892103E+05       94226.7       199.451     0.0828926      0.487678      0.222164      0.228942    -0.0247630    -0.0275191     0.0100105       1.50971       Opttag_D
      11       44.3407       2.44760       1.00000  0.000000E+00       1.00000  0.000000E+00      0.792596       68.4588       1.00000       24.6354  0.000000E+00       3.36265  0.000000E+00       3.36265  0.000000E+00  0.000000E+00      -6.72530       44.9909       1.47392        1       Scatag_D
      11       2825.27       12439.5  0.000000E+00  0.000000E+00       2371.02  0.000000E+00       5524.57       16138.9  0.000000E+00       18962.2       56.5972       35908.7 -1.000000E+10 -1.000000E+10 -1.000000E+10 -1.000000E+10 -1.000000E+10  0.000000E+00  0.000000E+00  0.000000E+00  0.000000E+00  0.000000E+00  0.000000E+00  0.000000E+00  0.000000E+00  0.000000E+00  0.000000E+00  0.000000E+00  0.000000E+00       Funtag_D
      12  2.785214E+05  1.892970E+05       89224.4       135.409     0.0858546      0.485349      0.222432      0.229303    -0.0252875    -0.0281473    0.00971617       1.45346       Opttag_D
      12       44.3145       2.44779       1.00000  0.000000E+00       1.00000  0.000000E+00      0.792676       68.2113       1.00000       24.6764  0.000000E+00       3.38002  0.000000E+00       3.38002  0.000000E+00  0.000000E+00      -6.76004       44.9909       1.47392        1       Scatag_D
      12       2661.60       11529.9  0.000000E+00  0.000000E+00       2370.25  0.000000E+00       5312.02       14197.6  0.000000E+00       18582.1       59.5356       34511.4 -1.000000E+10 -1.000000E+10 -1.000000E+10 -1.000000E+10 -1.000000E+10  0.000000E+00  0.000000E+00  0.000000E+00  0.000000E+00  0.000000E+00  0.000000E+00  0.000000E+00  0.000000E+00  0.000000E+00  0.000000E+00  0.000000E+00  0.000000E+00       Funtag_D
      13  2.749816E+05  1.892862E+05       85695.4       115.353     0.0866299      0.477474      0.222482      0.229373    -0.0252223    -0.0282259    0.00953500       1.43245       Opttag_D
      13       44.2974       2.44226       1.00000  0.000000E+00       1.00000  0.000000E+00      0.792656       68.0625       1.00000       24.6994  0.000000E+00       3.38897  0.000000E+00       3.38897  0.000000E+00  0.000000E+00      -6.77794       44.9909       1.47392        1       Scatag_D
      13       2563.27       11198.8  0.000000E+00  0.000000E+00       2367.93  0.000000E+00       5156.25       12710.3  0.000000E+00       18358.9       64.9928       33275.0 -1.000000E+10 -1.000000E+10 -1.000000E+10 -1.000000E+10 -1.000000E+10  0.000000E+00  0.000000E+00  0.000000E+00  0.000000E+00  0.000000E+00  0.000000E+00  0.000000E+00  0.000000E+00  0.000000E+00  0.000000E+00  0.000000E+00  0.000000E+00       Funtag_D
      14  2.711117E+05  1.893799E+05       81731.8       111.701     0.0898054      0.467284      0.222791      0.229756    -0.0257890    -0.0290936    0.00957065       1.41227       Opttag_D
      14       44.2615       2.44005       1.00000  0.000000E+00       1.00000  0.000000E+00      0.792662       67.7359       1.00000       24.7537  0.000000E+00       3.41398  0.000000E+00       3.41398  0.000000E+00  0.000000E+00      -6.82797       44.9909       1.47392        1       Scatag_D
      14       2582.47       10885.6  0.000000E+00  0.000000E+00       2364.81  0.000000E+00       4883.26       11705.9  0.000000E+00       17881.9       70.2484       31357.7 -1.000000E+10 -1.000000E+10 -1.000000E+10 -1.000000E+10 -1.000000E+10  0.000000E+00  0.000000E+00  0.000000E+00  0.000000E+00  0.000000E+00  0.000000E+00  0.000000E+00  0.000000E+00  0.000000E+00  0.000000E+00  0.000000E+00  0.000000E+00       Funtag_D
      15  2.700193E+05  1.896818E+05       80337.4       254.867     0.0966706      0.514522      0.223562      0.230750    -0.0276161    -0.0315180     0.0101853       1.52821       Opttag_D
      15       44.2073       2.44907       1.00000  0.000000E+00       1.00000  0.000000E+00      0.792745       67.1850       1.00000       24.8589  0.000000E+00       3.46180  0.000000E+00       3.46180  0.000000E+00  0.000000E+00      -6.92361       44.9909       1.47392        1       Scatag_D
      15       2924.83       12746.2  0.000000E+00  0.000000E+00       2363.93  0.000000E+00       4553.20       10954.7  0.000000E+00       17145.9       68.0103       29580.7 -1.000000E+10 -1.000000E+10 -1.000000E+10 -1.000000E+10 -1.000000E+10  0.000000E+00  0.000000E+00  0.000000E+00  0.000000E+00  0.000000E+00  0.000000E+00  0.000000E+00  0.000000E+00  0.000000E+00  0.000000E+00  0.000000E+00  0.000000E+00       Funtag_D
      16  2.662618E+05  1.896625E+05       76599.4       90.7113     0.0963979      0.508245      0.223517      0.230722    -0.0274990    -0.0314410    0.00941526       1.38643       Opttag_D
      16       44.2050       2.44698       1.00000  0.000000E+00       1.00000  0.000000E+00      0.792698       67.1921       1.00000       24.8576  0.000000E+00       3.46191  0.000000E+00       3.46191  0.000000E+00  0.000000E+00      -6.92381       44.9909       1.47392        1       Scatag_D
      16       2499.30       10490.9  0.000000E+00  0.000000E+00       2363.08  0.000000E+00       4556.41       10201.5  0.000000E+00       17126.2       63.0541       29299.0 -1.000000E+10 -1.000000E+10 -1.000000E+10 -1.000000E+10 -1.000000E+10  0.000000E+00  0.000000E+00  0.000000E+00  0.000000E+00  0.000000E+00  0.000000E+00  0.000000E+00  0.000000E+00  0.000000E+00  0.000000E+00  0.000000E+00  0.000000E+00       Funtag_D
      17  2.645041E+05  1.897242E+05       74779.8       71.8466     0.0978221      0.517071      0.223664      0.230941    -0.0278727    -0.0319864    0.00912345       1.37884       Opttag_D
      17       44.1926       2.44845       1.00000  0.000000E+00       1.00000  0.000000E+00      0.792681       67.0817       1.00000       24.8805  0.000000E+00       3.47208  0.000000E+00       3.47208  0.000000E+00  0.000000E+00      -6.94416       44.9909       1.47392        1       Scatag_D
      17       2346.77       10376.3  0.000000E+00  0.000000E+00       2362.77  0.000000E+00       4504.47       9433.42  0.000000E+00       16949.1       57.1113       28749.9 -1.000000E+10 -1.000000E+10 -1.000000E+10 -1.000000E+10 -1.000000E+10  0.000000E+00  0.000000E+00  0.000000E+00  0.000000E+00  0.000000E+00  0.000000E+00  0.000000E+00  0.000000E+00  0.000000E+00  0.000000E+00  0.000000E+00  0.000000E+00       Funtag_D
      18  2.622612E+05  1.899036E+05       72357.6       90.9192      0.101777      0.545345      0.224098      0.231521    -0.0289578    -0.0334601    0.00894673       1.38175       Opttag_D
      18       44.1659       2.45482       1.00000  0.000000E+00       1.00000  0.000000E+00      0.792699       66.7810       1.00000       24.9432  0.000000E+00       3.49658  0.000000E+00       3.49658  0.000000E+00  0.000000E+00      -6.99316       44.9909       1.47392        1       Scatag_D
      18       2256.74       10420.2  0.000000E+00  0.000000E+00       2362.70  0.000000E+00       4367.26       8705.50  0.000000E+00       16510.1       52.9862       27682.2 -1.000000E+10 -1.000000E+10 -1.000000E+10 -1.000000E+10 -1.000000E+10  0.000000E+00  0.000000E+00  0.000000E+00  0.000000E+00  0.000000E+00  0.000000E+00  0.000000E+00  0.000000E+00  0.000000E+00  0.000000E+00  0.000000E+00  0.000000E+00       Funtag_D
      19  2.596355E+05  1.901763E+05       69459.2       143.551      0.107660      0.570243      0.224748      0.232370    -0.0306083    -0.0356300    0.00889835       1.40637       Opttag_D
      19       44.1370       2.46639       1.00000  0.000000E+00       1.00000  0.000000E+00      0.792777       66.3255       1.00000       25.0385  0.000000E+00       3.53106  0.000000E+00       3.53106  0.000000E+00  0.000000E+00      -7.06212       44.9909       1.47392        1       Scatag_D
      19       2232.40       10794.8  0.000000E+00  0.000000E+00       2361.77  0.000000E+00       4178.18       7882.24  0.000000E+00       15833.3       54.3545       26122.2 -1.000000E+10 -1.000000E+10 -1.000000E+10 -1.000000E+10 -1.000000E+10  0.000000E+00  0.000000E+00  0.000000E+00  0.000000E+00  0.000000E+00  0.000000E+00  0.000000E+00  0.000000E+00  0.000000E+00  0.000000E+00  0.000000E+00  0.000000E+00       Funtag_D
      20  2.574264E+05  1.905747E+05       66851.7       115.565      0.114872      0.618348      0.225637      0.233477    -0.0330194    -0.0385404    0.00887890       1.37807       Opttag_D
      20       44.1175       2.48954       1.00000  0.000000E+00       1.00000  0.000000E+00      0.792979       65.8192       1.00000       25.1454  0.000000E+00       3.57252  0.000000E+00       3.57252  0.000000E+00  0.000000E+00      -7.14504       44.9909       1.47392        1       Scatag_D
      20       2222.65       10364.7  0.000000E+00  0.000000E+00       2363.70  0.000000E+00       4012.20       7433.99  0.000000E+00       15141.3       60.6680       25252.5 -1.000000E+10 -1.000000E+10 -1.000000E+10 -1.000000E+10 -1.000000E+10  0.000000E+00  0.000000E+00  0.000000E+00  0.000000E+00  0.000000E+00  0.000000E+00  0.000000E+00  0.000000E+00  0.000000E+00  0.000000E+00  0.000000E+00  0.000000E+00       Funtag_D
      21  2.557186E+05  1.905297E+05       65188.9       62.7072      0.114287      0.615174      0.225547      0.233379    -0.0327468    -0.0382644    0.00858999       1.34995       Opttag_D
      21       44.1212       2.48652       1.00000  0.000000E+00       1.00000  0.000000E+00      0.792980       65.8275       1.00000       25.1306  0.000000E+00       3.57316  0.000000E+00       3.57316  0.000000E+00  0.000000E+00      -7.14633       44.9909       1.47392        1       Scatag_D
      21       2080.36       9946.09  0.000000E+00  0.000000E+00       2361.44  0.000000E+00       3986.30       6878.02  0.000000E+00       15087.0       62.4351       24787.2 -1.000000E+10 -1.000000E+10 -1.000000E+10 -1.000000E+10 -1.000000E+10  0.000000E+00  0.000000E+00  0.000000E+00  0.000000E+00  0.000000E+00  0.000000E+00  0.000000E+00  0.000000E+00  0.000000E+00  0.000000E+00  0.000000E+00  0.000000E+00       Funtag_D
      22  2.538656E+05  1.905556E+05       63310.0       64.9493      0.115049      0.636246      0.225613      0.233489    -0.0329035    -0.0385787    0.00844578       1.34151       Opttag_D
      22       44.1259       2.48835       1.00000  0.000000E+00       1.00000  0.000000E+00      0.793039       65.7303       1.00000       25.1241  0.000000E+00       3.58438  0.000000E+00       3.58438  0.000000E+00  0.000000E+00      -7.16877       44.9909       1.47392        1       Scatag_D
      22       2011.09       9822.01  0.000000E+00  0.000000E+00       2357.63  0.000000E+00       3911.67       6391.10  0.000000E+00       14831.1       66.1890       23919.2 -1.000000E+10 -1.000000E+10 -1.000000E+10 -1.000000E+10 -1.000000E+10  0.000000E+00  0.000000E+00  0.000000E+00  0.000000E+00  0.000000E+00  0.000000E+00  0.000000E+00  0.000000E+00  0.000000E+00  0.000000E+00  0.000000E+00  0.000000E+00       Funtag_D
      23  2.525505E+05  1.906971E+05       61853.5       88.7080      0.117898      0.681609      0.225929      0.233910    -0.0337596    -0.0398391    0.00846259       1.34260       Opttag_D
      23       44.1258       2.49778       1.00000  0.000000E+00       1.00000  0.000000E+00      0.793125       65.5250       1.00000       25.1485  0.000000E+00       3.60253  0.000000E+00       3.60253  0.000000E+00  0.000000E+00      -7.20507       44.9909       1.47392        1       Scatag_D
      23       2019.11       9838.09  0.000000E+00  0.000000E+00       2356.16  0.000000E+00       3833.31       6055.25  0.000000E+00       14461.6       63.2918       23226.6 -1.000000E+10 -1.000000E+10 -1.000000E+10 -1.000000E+10 -1.000000E+10  0.000000E+00  0.000000E+00  0.000000E+00  0.000000E+00  0.000000E+00  0.000000E+00  0.000000E+00  0.000000E+00  0.000000E+00  0.000000E+00  0.000000E+00  0.000000E+00       Funtag_D
      24  2.509286E+05  1.909160E+05       60012.6       61.4086      0.122335      0.745804      0.226427      0.234581    -0.0350846    -0.0418185    0.00840495       1.31977       Opttag_D
      24       44.1266       2.51284       1.00000  0.000000E+00       1.00000  0.000000E+00      0.793227       65.2340       1.00000       25.1976  0.000000E+00       3.62426  0.000000E+00       3.62426  0.000000E+00  0.000000E+00      -7.24851       44.9909       1.47392        1       Scatag_D
      24       1991.69       9506.32  0.000000E+00  0.000000E+00       2355.35  0.000000E+00       3760.36       5681.75  0.000000E+00       13997.9       55.3825       22663.9 -1.000000E+10 -1.000000E+10 -1.000000E+10 -1.000000E+10 -1.000000E+10  0.000000E+00  0.000000E+00  0.000000E+00  0.000000E+00  0.000000E+00  0.000000E+00  0.000000E+00  0.000000E+00  0.000000E+00  0.000000E+00  0.000000E+00  0.000000E+00       Funtag_D
      25  2.495320E+05  1.909993E+05       58532.6       62.6659      0.125152      0.788905      0.226654      0.234958    -0.0355887    -0.0429484    0.00820189       1.31182       Opttag_D
      25       44.1262       2.51753       1.00000  0.000000E+00       1.00000  0.000000E+00      0.793202       65.0512       1.00000       25.2390  0.000000E+00       3.62943  0.000000E+00       3.62943  0.000000E+00  0.000000E+00      -7.25887       44.9909       1.47392        1       Scatag_D
      25       1896.62       9392.13  0.000000E+00  0.000000E+00       2355.41  0.000000E+00       3727.96       5279.23  0.000000E+00       13699.5       48.3910       22133.4 -1.000000E+10 -1.000000E+10 -1.000000E+10 -1.000000E+10 -1.000000E+10  0.000000E+00  0.000000E+00  0.000000E+00  0.000000E+00  0.000000E+00  0.000000E+00  0.000000E+00  0.000000E+00  0.000000E+00  0.000000E+00  0.000000E+00  0.000000E+00       Funtag_D
      26  2.481485E+05  1.910877E+05       57060.7       74.4826      0.129180      0.849168      0.226923      0.235487    -0.0361238    -0.0444107    0.00804718       1.30319       Opttag_D
      26       44.1299       2.52240       1.00000  0.000000E+00       1.00000  0.000000E+00      0.793141       64.8083       1.00000       25.3036  0.000000E+00       3.62944  0.000000E+00       3.62944  0.000000E+00  0.000000E+00      -7.25888       44.9909       1.47392        1       Scatag_D
      26       1825.74       9268.93  0.000000E+00  0.000000E+00       2356.42  0.000000E+00       3711.27       5123.36  0.000000E+00       13321.4       43.7880       21409.8 -1.000000E+10 -1.000000E+10 -1.000000E+10 -1.000000E+10 -1.000000E+10  0.000000E+00  0.000000E+00  0.000000E+00  0.000000E+00  0.000000E+00  0.000000E+00  0.000000E+00  0.000000E+00  0.000000E+00  0.000000E+00  0.000000E+00  0.000000E+00       Funtag_D
      27  2.470561E+05  1.910645E+05       55991.6       55.8827      0.130523      0.872199      0.226920      0.235601    -0.0359831    -0.0446374    0.00791828       1.29012       Opttag_D
      27       44.1368       2.52033       1.00000  0.000000E+00       1.00000  0.000000E+00      0.793099       64.7291       1.00000       25.3288  0.000000E+00       3.62367  0.000000E+00       3.62367  0.000000E+00  0.000000E+00      -7.24734       44.9909       1.47392        1       Scatag_D
      27       1767.73       9083.97  0.000000E+00  0.000000E+00       2356.40  0.000000E+00       3709.79       4903.15  0.000000E+00       13170.5       46.3485       20953.8 -1.000000E+10 -1.000000E+10 -1.000000E+10 -1.000000E+10 -1.000000E+10  0.000000E+00  0.000000E+00  0.000000E+00  0.000000E+00  0.000000E+00  0.000000E+00  0.000000E+00  0.000000E+00  0.000000E+00  0.000000E+00  0.000000E+00  0.000000E+00       Funtag_D
      28  2.458896E+05  1.910318E+05       54857.8       56.2117      0.132192      0.900119      0.226894      0.235719    -0.0357852    -0.0448160    0.00779412       1.28194       Opttag_D
      28       44.1513       2.51848       1.00000  0.000000E+00       1.00000  0.000000E+00      0.793099       64.6394       1.00000       25.3589  0.000000E+00       3.61660  0.000000E+00       3.61660  0.000000E+00  0.000000E+00      -7.23320       44.9909       1.47392        1       Scatag_D
      28       1712.72       8969.08  0.000000E+00  0.000000E+00       2356.06  0.000000E+00       3704.85       4693.26  0.000000E+00       12978.5       53.0730       20390.3 -1.000000E+10 -1.000000E+10 -1.000000E+10 -1.000000E+10 -1.000000E+10  0.000000E+00  0.000000E+00  0.000000E+00  0.000000E+00  0.000000E+00  0.000000E+00  0.000000E+00  0.000000E+00  0.000000E+00  0.000000E+00  0.000000E+00  0.000000E+00       Funtag_D
      29  2.450164E+05  1.911015E+05       53914.9       93.8471      0.135818      0.951314      0.227061      0.236083    -0.0362069    -0.0457636    0.00784631       1.28667       Opttag_D
      29       44.1833       2.52734       1.00000  0.000000E+00       1.00000  0.000000E+00      0.793251       64.4770       1.00000       25.4074  0.000000E+00       3.61492  0.000000E+00       3.61492  0.000000E+00  0.000000E+00      -7.22984       44.9909       1.47392        1       Scatag_D
      29       1735.73       9035.45  0.000000E+00  0.000000E+00       2356.31  0.000000E+00       3686.98       4621.94  0.000000E+00       12661.8       60.1430       19756.5 -1.000000E+10 -1.000000E+10 -1.000000E+10 -1.000000E+10 -1.000000E+10  0.000000E+00  0.000000E+00  0.000000E+00  0.000000E+00  0.000000E+00  0.000000E+00  0.000000E+00  0.000000E+00  0.000000E+00  0.000000E+00  0.000000E+00  0.000000E+00       Funtag_D
      30  2.440362E+05  1.911762E+05       52860.0       50.8217      0.138031      0.980221      0.227218      0.236333    -0.0366589    -0.0464902    0.00774341       1.26269       Opttag_D
      30       44.2027       2.53553       1.00000  0.000000E+00       1.00000  0.000000E+00      0.793386       64.3769       1.00000       25.4400  0.000000E+00       3.61645  0.000000E+00       3.61645  0.000000E+00  0.000000E+00      -7.23290       44.9909       1.47392        1       Scatag_D
      30       1690.51       8701.74  0.000000E+00  0.000000E+00       2355.72  0.000000E+00       3666.97       4471.07  0.000000E+00       12492.8       58.6417       19422.6 -1.000000E+10 -1.000000E+10 -1.000000E+10 -1.000000E+10 -1.000000E+10  0.000000E+00  0.000000E+00  0.000000E+00  0.000000E+00  0.000000E+00  0.000000E+00  0.000000E+00  0.000000E+00  0.000000E+00  0.000000E+00  0.000000E+00  0.000000E+00       Funtag_D
      31  2.432381E+05  1.912753E+05       51962.8       47.2901      0.140105       1.00616      0.227417      0.236591    -0.0372586    -0.0472996    0.00771959       1.25830       Opttag_D
      31       44.2212       2.54589       1.00000  0.000000E+00       1.00000  0.000000E+00      0.793559       64.2838       1.00000       25.4685  0.000000E+00       3.62111  0.000000E+00       3.62111  0.000000E+00  0.000000E+00      -7.24222       44.9909       1.47392        1       Scatag_D
      31       1680.12       8641.45  0.000000E+00  0.000000E+00       2355.03  0.000000E+00       3629.55       4221.42  0.000000E+00       12341.2       52.3127       19041.8 -1.000000E+10 -1.000000E+10 -1.000000E+10 -1.000000E+10 -1.000000E+10  0.000000E+00  0.000000E+00  0.000000E+00  0.000000E+00  0.000000E+00  0.000000E+00  0.000000E+00  0.000000E+00  0.000000E+00  0.000000E+00  0.000000E+00  0.000000E+00       Funtag_D
      32  2.424297E+05  1.914104E+05       51019.3       51.3377      0.143129       1.04407      0.227683      0.236936    -0.0380763    -0.0484257    0.00768324       1.25439       Opttag_D
      32       44.2488       2.56043       1.00000  0.000000E+00       1.00000  0.000000E+00      0.793811       64.1511       1.00000       25.5081  0.000000E+00       3.62527  0.000000E+00       3.62527  0.000000E+00  0.000000E+00      -7.25054       44.9909       1.47392        1       Scatag_D
      32       1664.34       8587.80  0.000000E+00  0.000000E+00       2353.78  0.000000E+00       3580.60       4036.50  0.000000E+00       12128.3       45.0822       18622.9 -1.000000E+10 -1.000000E+10 -1.000000E+10 -1.000000E+10 -1.000000E+10  0.000000E+00  0.000000E+00  0.000000E+00  0.000000E+00  0.000000E+00  0.000000E+00  0.000000E+00  0.000000E+00  0.000000E+00  0.000000E+00  0.000000E+00  0.000000E+00       Funtag_D
      33  2.423001E+05  1.915845E+05       50715.6       125.360      0.147795       1.10269      0.228022      0.237398    -0.0391300    -0.0499684    0.00777972       1.28707       Opttag_D
      33       44.2947       2.58077       1.00000  0.000000E+00       1.00000  0.000000E+00      0.794202       63.9550       1.00000       25.5673  0.000000E+00       3.62595  0.000000E+00       3.62595  0.000000E+00  0.000000E+00      -7.25189       44.9909       1.47392        1       Scatag_D
      33       1706.40       9041.02  0.000000E+00  0.000000E+00       2351.73  0.000000E+00       3521.74       3890.70  0.000000E+00       11819.6       41.4295       18342.9 -1.000000E+10 -1.000000E+10 -1.000000E+10 -1.000000E+10 -1.000000E+10  0.000000E+00  0.000000E+00  0.000000E+00  0.000000E+00  0.000000E+00  0.000000E+00  0.000000E+00  0.000000E+00  0.000000E+00  0.000000E+00  0.000000E+00  0.000000E+00       Funtag_D
      34  2.413814E+05  1.915416E+05       49839.8       38.2130      0.146980       1.09389      0.227935      0.237293    -0.0388704    -0.0496389    0.00750101       1.24299       Opttag_D
      34       44.2880       2.57641       1.00000  0.000000E+00       1.00000  0.000000E+00      0.794154       63.9872       1.00000       25.5511  0.000000E+00       3.62481  0.000000E+00       3.62481  0.000000E+00  0.000000E+00      -7.24962       44.9909       1.47392        1       Scatag_D
      34       1586.33       8432.44  0.000000E+00  0.000000E+00       2351.53  0.000000E+00       3519.86       3833.70  0.000000E+00       11848.3       42.3372       18225.3 -1.000000E+10 -1.000000E+10 -1.000000E+10 -1.000000E+10 -1.000000E+10  0.000000E+00  0.000000E+00  0.000000E+00  0.000000E+00  0.000000E+00  0.000000E+00  0.000000E+00  0.000000E+00  0.000000E+00  0.000000E+00  0.000000E+00  0.000000E+00       Funtag_D
      35  2.410608E+05  1.915249E+05       49535.9       27.5539      0.147134       1.09742      0.227893      0.237276    -0.0387695    -0.0495822    0.00743379       1.24002       Opttag_D
      35       44.2920       2.57563       1.00000  0.000000E+00       1.00000  0.000000E+00      0.794181       63.9812       1.00000       25.5467  0.000000E+00       3.62264  0.000000E+00       3.62264  0.000000E+00  0.000000E+00      -7.24527       44.9909       1.47392        1       Scatag_D
      35       1558.02       8392.14  0.000000E+00  0.000000E+00       2350.86  0.000000E+00       3511.10       3771.48  0.000000E+00       11816.1       44.3584       18091.8 -1.000000E+10 -1.000000E+10 -1.000000E+10 -1.000000E+10 -1.000000E+10  0.000000E+00  0.000000E+00  0.000000E+00  0.000000E+00  0.000000E+00  0.000000E+00  0.000000E+00  0.000000E+00  0.000000E+00  0.000000E+00  0.000000E+00  0.000000E+00       Funtag_D
      36  2.405500E+05  1.915417E+05       49008.2       39.0986      0.148748       1.12045      0.227905      0.237365    -0.0388711    -0.0499096    0.00739115       1.23797       Opttag_D
      36       44.3154       2.58044       1.00000  0.000000E+00       1.00000  0.000000E+00      0.794361       63.9210       1.00000       25.5530  0.000000E+00       3.61843  0.000000E+00       3.61843  0.000000E+00  0.000000E+00      -7.23687       44.9909       1.47392        1       Scatag_D
      36       1540.20       8364.37  0.000000E+00  0.000000E+00       2349.00  0.000000E+00       3484.52       3726.43  0.000000E+00       11674.5       47.9009       17821.3 -1.000000E+10 -1.000000E+10 -1.000000E+10 -1.000000E+10 -1.000000E+10  0.000000E+00  0.000000E+00  0.000000E+00  0.000000E+00  0.000000E+00  0.000000E+00  0.000000E+00  0.000000E+00  0.000000E+00  0.000000E+00  0.000000E+00  0.000000E+00       Funtag_D
      37  2.398939E+05  1.916021E+05       48291.8       65.5667      0.151898       1.16340      0.227971      0.237572    -0.0392362    -0.0507156    0.00742100       1.23828       Opttag_D
      37       44.3645       2.59292       1.00000  0.000000E+00       1.00000  0.000000E+00      0.794742       63.8122       1.00000       25.5695  0.000000E+00       3.61267  0.000000E+00       3.61267  0.000000E+00  0.000000E+00      -7.22535       44.9909       1.47392        1       Scatag_D
      37       1552.67       8368.61  0.000000E+00  0.000000E+00       2345.61  0.000000E+00       3446.13       3634.10  0.000000E+00       11430.6       48.8396       17465.2 -1.000000E+10 -1.000000E+10 -1.000000E+10 -1.000000E+10 -1.000000E+10  0.000000E+00  0.000000E+00  0.000000E+00  0.000000E+00  0.000000E+00  0.000000E+00  0.000000E+00  0.000000E+00  0.000000E+00  0.000000E+00  0.000000E+00  0.000000E+00       Funtag_D
      38  2.392935E+05  1.917400E+05       47553.5       52.6069      0.157073       1.22941      0.228180      0.238045    -0.0400709    -0.0522093    0.00749228       1.22213       Opttag_D
      38       44.4434       2.61654       1.00000  0.000000E+00       1.00000  0.000000E+00      0.795354       63.6417       1.00000       25.6107  0.000000E+00       3.60472  0.000000E+00       3.60472  0.000000E+00  0.000000E+00      -7.20944       44.9909       1.47392        1       Scatag_D
      38       1582.64       8151.72  0.000000E+00  0.000000E+00       2342.64  0.000000E+00       3412.70       3570.04  0.000000E+00       11109.6       48.0986       17336.1 -1.000000E+10 -1.000000E+10 -1.000000E+10 -1.000000E+10 -1.000000E+10  0.000000E+00  0.000000E+00  0.000000E+00  0.000000E+00  0.000000E+00  0.000000E+00  0.000000E+00  0.000000E+00  0.000000E+00  0.000000E+00  0.000000E+00  0.000000E+00       Funtag_D
      39  2.387812E+05  1.917727E+05       47008.5       29.6422      0.158013       1.24207      0.228232      0.238156    -0.0402690    -0.0525600    0.00742520       1.21150       Opttag_D
      39       44.4612       2.62195       1.00000  0.000000E+00       1.00000  0.000000E+00      0.795481       63.6075       1.00000       25.6210  0.000000E+00       3.60428  0.000000E+00       3.60428  0.000000E+00  0.000000E+00      -7.20856       44.9909       1.47392        1       Scatag_D
      39       1554.42       8010.53  0.000000E+00  0.000000E+00       2341.47  0.000000E+00       3397.53       3429.89  0.000000E+00       11048.6       44.7597       17181.2 -1.000000E+10 -1.000000E+10 -1.000000E+10 -1.000000E+10 -1.000000E+10  0.000000E+00  0.000000E+00  0.000000E+00  0.000000E+00  0.000000E+00  0.000000E+00  0.000000E+00  0.000000E+00  0.000000E+00  0.000000E+00  0.000000E+00  0.000000E+00       Funtag_D
      40  2.382722E+05  1.918295E+05       46442.7       36.5852      0.159596       1.26385      0.228318      0.238342    -0.0406127    -0.0531702    0.00744207       1.20537       Opttag_D
      40       44.4930       2.63161       1.00000  0.000000E+00       1.00000  0.000000E+00      0.795696       63.5527       1.00000       25.6367  0.000000E+00       3.60342  0.000000E+00       3.60342  0.000000E+00  0.000000E+00      -7.20685       44.9909       1.47392        1       Scatag_D
      40       1561.49       7929.64  0.000000E+00  0.000000E+00       2339.21  0.000000E+00       3372.11       3347.12  0.000000E+00       10947.5       39.5372       16906.0 -1.000000E+10 -1.000000E+10 -1.000000E+10 -1.000000E+10 -1.000000E+10  0.000000E+00  0.000000E+00  0.000000E+00  0.000000E+00  0.000000E+00  0.000000E+00  0.000000E+00  0.000000E+00  0.000000E+00  0.000000E+00  0.000000E+00  0.000000E+00       Funtag_D
      41  2.379191E+05  1.919119E+05       46007.2       43.7709      0.162072       1.29660      0.228443      0.238620    -0.0411113    -0.0540492    0.00746458       1.19815       Opttag_D
      41       44.5386       2.64563       1.00000  0.000000E+00       1.00000  0.000000E+00      0.796009       63.4730       1.00000       25.6583  0.000000E+00       3.60062  0.000000E+00       3.60062  0.000000E+00  0.000000E+00      -7.20124       44.9909       1.47392        1       Scatag_D
      41       1570.95       7834.98  0.000000E+00  0.000000E+00       2336.41  0.000000E+00       3352.37       3358.43  0.000000E+00       10809.2       37.0227       16707.9 -1.000000E+10 -1.000000E+10 -1.000000E+10 -1.000000E+10 -1.000000E+10  0.000000E+00  0.000000E+00  0.000000E+00  0.000000E+00  0.000000E+00  0.000000E+00  0.000000E+00  0.000000E+00  0.000000E+00  0.000000E+00  0.000000E+00  0.000000E+00       Funtag_D
      42  2.374506E+05  1.919922E+05       45458.4       29.8108      0.164679       1.33097      0.228571      0.238911    -0.0415972    -0.0549280    0.00741014       1.18641       Opttag_D
      42       44.5864       2.65991       1.00000  0.000000E+00       1.00000  0.000000E+00      0.796320       63.3928       1.00000       25.6793  0.000000E+00       3.59690  0.000000E+00       3.59690  0.000000E+00  0.000000E+00      -7.19380       44.9909       1.47392        1       Scatag_D
      42       1548.12       7682.26  0.000000E+00  0.000000E+00       2333.16  0.000000E+00       3340.33       3285.55  0.000000E+00       10672.8       37.3732       16558.8 -1.000000E+10 -1.000000E+10 -1.000000E+10 -1.000000E+10 -1.000000E+10  0.000000E+00  0.000000E+00  0.000000E+00  0.000000E+00  0.000000E+00  0.000000E+00  0.000000E+00  0.000000E+00  0.000000E+00  0.000000E+00  0.000000E+00  0.000000E+00       Funtag_D
      43  2.369184E+05  1.920565E+05       44861.9       35.9851      0.167353       1.36804      0.228660      0.239229    -0.0419863    -0.0558018    0.00733144       1.18273       Opttag_D
      43       44.6403       2.67383       1.00000  0.000000E+00       1.00000  0.000000E+00      0.796623       63.3157       1.00000       25.6950  0.000000E+00       3.59155  0.000000E+00       3.59155  0.000000E+00  0.000000E+00      -7.18310       44.9909       1.47392        1       Scatag_D
      43       1515.41       7634.66  0.000000E+00  0.000000E+00       2328.30  0.000000E+00       3334.19       3131.87  0.000000E+00       10527.7       41.3414       16348.5 -1.000000E+10 -1.000000E+10 -1.000000E+10 -1.000000E+10 -1.000000E+10  0.000000E+00  0.000000E+00  0.000000E+00  0.000000E+00  0.000000E+00  0.000000E+00  0.000000E+00  0.000000E+00  0.000000E+00  0.000000E+00  0.000000E+00  0.000000E+00       Funtag_D
      44  2.367127E+05  1.920934E+05       44619.3       64.7742      0.169473       1.39903      0.228696      0.239473    -0.0422093    -0.0564765    0.00738524       1.18887       Opttag_D
      44       44.6898       2.68503       1.00000  0.000000E+00       1.00000  0.000000E+00      0.796859       63.2672       1.00000       25.6999  0.000000E+00       3.58603  0.000000E+00       3.58603  0.000000E+00  0.000000E+00      -7.17205       44.9909       1.47392        1       Scatag_D
      44       1537.74       7714.03  0.000000E+00  0.000000E+00       2323.67  0.000000E+00       3335.00       3110.73  0.000000E+00       10409.4       45.2110       16143.6 -1.000000E+10 -1.000000E+10 -1.000000E+10 -1.000000E+10 -1.000000E+10  0.000000E+00  0.000000E+00  0.000000E+00  0.000000E+00  0.000000E+00  0.000000E+00  0.000000E+00  0.000000E+00  0.000000E+00  0.000000E+00  0.000000E+00  0.000000E+00       Funtag_D
      45  2.363673E+05  1.920534E+05       44314.0       27.7227      0.168645       1.39014      0.228624      0.239372    -0.0419671    -0.0561780    0.00726675       1.17583       Opttag_D
      45       44.6791       2.67992       1.00000  0.000000E+00       1.00000  0.000000E+00      0.796761       63.2943       1.00000       25.6893  0.000000E+00       3.58638  0.000000E+00       3.58638  0.000000E+00  0.000000E+00      -7.17277       44.9909       1.47392        1       Scatag_D
      45       1488.79       7545.76  0.000000E+00  0.000000E+00       2323.95  0.000000E+00       3332.33       3080.47  0.000000E+00       10432.2       44.5192       16066.0 -1.000000E+10 -1.000000E+10 -1.000000E+10 -1.000000E+10 -1.000000E+10  0.000000E+00  0.000000E+00  0.000000E+00  0.000000E+00  0.000000E+00  0.000000E+00  0.000000E+00  0.000000E+00  0.000000E+00  0.000000E+00  0.000000E+00  0.000000E+00       Funtag_D
      46  2.361057E+05  1.920371E+05       44068.6       22.2742      0.168773       1.39455      0.228579      0.239376    -0.0418685    -0.0562054    0.00722966       1.17193       Opttag_D
      46       44.6905       2.68050       1.00000  0.000000E+00       1.00000  0.000000E+00      0.796780       63.3002       1.00000       25.6850  0.000000E+00       3.58469  0.000000E+00       3.58469  0.000000E+00  0.000000E+00      -7.16939       44.9909       1.47392        1       Scatag_D
      46       1473.63       7495.85  0.000000E+00  0.000000E+00       2322.68  0.000000E+00       3329.39       3041.82  0.000000E+00       10408.2       42.7370       15954.4 -1.000000E+10 -1.000000E+10 -1.000000E+10 -1.000000E+10 -1.000000E+10  0.000000E+00  0.000000E+00  0.000000E+00  0.000000E+00  0.000000E+00  0.000000E+00  0.000000E+00  0.000000E+00  0.000000E+00  0.000000E+00  0.000000E+00  0.000000E+00       Funtag_D
      47  2.358006E+05  1.920385E+05       43762.2       29.5481      0.169893       1.41348      0.228552      0.239481    -0.0418770    -0.0565569    0.00724213       1.16715       Opttag_D
      47       44.7276       2.68687       1.00000  0.000000E+00       1.00000  0.000000E+00      0.796919       63.2890       1.00000       25.6859  0.000000E+00       3.58014  0.000000E+00       3.58014  0.000000E+00  0.000000E+00      -7.16029       44.9909       1.47392        1       Scatag_D
      47       1478.72       7434.80  0.000000E+00  0.000000E+00       2319.85  0.000000E+00       3326.35       3011.10  0.000000E+00       10333.5       40.3672       15817.4 -1.000000E+10 -1.000000E+10 -1.000000E+10 -1.000000E+10 -1.000000E+10  0.000000E+00  0.000000E+00  0.000000E+00  0.000000E+00  0.000000E+00  0.000000E+00  0.000000E+00  0.000000E+00  0.000000E+00  0.000000E+00  0.000000E+00  0.000000E+00       Funtag_D
      48  2.355101E+05  1.920671E+05       43443.0       54.9987      0.172329       1.45143      0.228547      0.239702    -0.0420500    -0.0572933    0.00726412       1.16637       Opttag_D
      48       44.7985       2.70127       1.00000  0.000000E+00       1.00000  0.000000E+00      0.797212       63.2610       1.00000       25.6964  0.000000E+00       3.57101  0.000000E+00       3.57101  0.000000E+00  0.000000E+00      -7.14202       44.9909       1.47392        1       Scatag_D
      48       1487.71       7424.81  0.000000E+00  0.000000E+00       2316.14  0.000000E+00       3321.08       2939.52  0.000000E+00       10200.6       39.6630       15713.5 -1.000000E+10 -1.000000E+10 -1.000000E+10 -1.000000E+10 -1.000000E+10  0.000000E+00  0.000000E+00  0.000000E+00  0.000000E+00  0.000000E+00  0.000000E+00  0.000000E+00  0.000000E+00  0.000000E+00  0.000000E+00  0.000000E+00  0.000000E+00       Funtag_D
      49  2.351735E+05  1.920994E+05       43074.1       22.4394      0.174320       1.48090      0.228564      0.239896    -0.0422459    -0.0579241    0.00721626       1.15027       Opttag_D
      49       44.8534       2.71334       1.00000  0.000000E+00       1.00000  0.000000E+00      0.797449       63.2364       1.00000       25.7090  0.000000E+00       3.56404  0.000000E+00       3.56404  0.000000E+00  0.000000E+00      -7.12808       44.9909       1.47392        1       Scatag_D
      49       1468.17       7221.34  0.000000E+00  0.000000E+00       2313.66  0.000000E+00       3319.61       2905.66  0.000000E+00       10107.7       39.0295       15698.9 -1.000000E+10 -1.000000E+10 -1.000000E+10 -1.000000E+10 -1.000000E+10  0.000000E+00  0.000000E+00  0.000000E+00  0.000000E+00  0.000000E+00  0.000000E+00  0.000000E+00  0.000000E+00  0.000000E+00  0.000000E+00  0.000000E+00  0.000000E+00       Funtag_D

 An atom has moved more than     1.50 A from the initial/last
 position - if this was an accepted trial point, probably need to update the
 non-bonded contact list.

      50  2.349716E+05  1.921324E+05       42839.2       19.7793      0.175777       1.50130      0.228600      0.240034    -0.0424454    -0.0583678    0.00717825       1.14594       Opttag_D
      50       44.8898       2.72229       1.00000  0.000000E+00       1.00000  0.000000E+00      0.797616       63.2148       1.00000       25.7222  0.000000E+00       3.55931  0.000000E+00       3.55931  0.000000E+00  0.000000E+00      -7.11862       44.9909       1.47392        1       Scatag_D
      50       1452.75       7167.08  0.000000E+00  0.000000E+00       2312.25  0.000000E+00       3315.05       2883.51  0.000000E+00       10047.9       39.4151       15621.2 -1.000000E+10 -1.000000E+10 -1.000000E+10 -1.000000E+10 -1.000000E+10  0.000000E+00  0.000000E+00  0.000000E+00  0.000000E+00  0.000000E+00  0.000000E+00  0.000000E+00  0.000000E+00  0.000000E+00  0.000000E+00  0.000000E+00  0.000000E+00       Funtag_D

 An atom has moved more than     1.50 A from the initial/last
 position - need to update the non-bonded contact list.


 ############################################################################################################
 ###################################################### setting up contact terms
 ############################################################################################################

      51  2.347124E+05  1.921958E+05       42516.6       29.6456      0.178420       1.53735      0.228675      0.240291    -0.0428288    -0.0591745    0.00715968       1.14135       Opttag_D
      51       44.9548       2.73868       1.00000  0.000000E+00       1.00000  0.000000E+00      0.797916       63.1743       1.00000       25.7490  0.000000E+00       3.55095  0.000000E+00       3.55095  0.000000E+00  0.000000E+00      -7.10190       44.9909       1.47392        1       Scatag_D
      51       1445.24       7109.68  0.000000E+00  0.000000E+00       2309.62  0.000000E+00       3307.04       2876.54  0.000000E+00       9947.53       40.0525       15480.9 -1.000000E+10 -1.000000E+10 -1.000000E+10 -1.000000E+10 -1.000000E+10  0.000000E+00  0.000000E+00  0.000000E+00  0.000000E+00  0.000000E+00  0.000000E+00  0.000000E+00  0.000000E+00  0.000000E+00  0.000000E+00  0.000000E+00  0.000000E+00       Funtag_D
      52  2.345197E+05  1.923191E+05       42200.6       59.9133      0.183410       1.60304      0.228830      0.240776    -0.0435753    -0.0607034    0.00726098       1.13656       Opttag_D
      52       45.0777       2.77016       1.00000  0.000000E+00       1.00000  0.000000E+00      0.798471       63.0982       1.00000       25.8082  0.000000E+00       3.53534  0.000000E+00       3.53534  0.000000E+00  0.000000E+00      -7.07068       44.9909       1.47392        1       Scatag_D
      52       1486.42       7050.15  0.000000E+00  0.000000E+00       2304.66  0.000000E+00       3296.10       2935.46  0.000000E+00       9780.42       39.6573       15307.7 -1.000000E+10 -1.000000E+10 -1.000000E+10 -1.000000E+10 -1.000000E+10  0.000000E+00  0.000000E+00  0.000000E+00  0.000000E+00  0.000000E+00  0.000000E+00  0.000000E+00  0.000000E+00  0.000000E+00  0.000000E+00  0.000000E+00  0.000000E+00       Funtag_D
      53  2.341805E+05  1.923410E+05       41839.5       27.5324      0.184258       1.61356      0.228858      0.240847    -0.0437075    -0.0609506    0.00723383       1.12814       Opttag_D
      53       45.0994       2.77565       1.00000  0.000000E+00       1.00000  0.000000E+00      0.798561       63.0825       1.00000       25.8198  0.000000E+00       3.53301  0.000000E+00       3.53301  0.000000E+00  0.000000E+00      -7.06601       44.9909       1.47392        1       Scatag_D
      53       1475.33       6946.08  0.000000E+00  0.000000E+00       2303.22  0.000000E+00       3287.77       2807.23  0.000000E+00       9750.41       39.1441       15230.4 -1.000000E+10 -1.000000E+10 -1.000000E+10 -1.000000E+10 -1.000000E+10  0.000000E+00  0.000000E+00  0.000000E+00  0.000000E+00  0.000000E+00  0.000000E+00  0.000000E+00  0.000000E+00  0.000000E+00  0.000000E+00  0.000000E+00  0.000000E+00       Funtag_D
      54  2.339195E+05  1.923736E+05       41545.9       21.4967      0.185672       1.63102      0.228896      0.240962    -0.0439051    -0.0613657    0.00724127       1.12237       Opttag_D
      54       45.1372       2.78484       1.00000  0.000000E+00       1.00000  0.000000E+00      0.798709       63.0582       1.00000       25.8396  0.000000E+00       3.52884  0.000000E+00       3.52884  0.000000E+00  0.000000E+00      -7.05767       44.9909       1.47392        1       Scatag_D
      54       1478.37       6875.29  0.000000E+00  0.000000E+00       2300.47  0.000000E+00       3272.80       2766.61  0.000000E+00       9698.52       38.1823       15115.7 -1.000000E+10 -1.000000E+10 -1.000000E+10 -1.000000E+10 -1.000000E+10  0.000000E+00  0.000000E+00  0.000000E+00  0.000000E+00  0.000000E+00  0.000000E+00  0.000000E+00  0.000000E+00  0.000000E+00  0.000000E+00  0.000000E+00  0.000000E+00       Funtag_D
      55  2.337009E+05  1.924333E+05       41267.6       24.8205      0.187953       1.65901      0.228972      0.241153    -0.0442663    -0.0620702    0.00726751       1.11686       Opttag_D
      55       45.1968       2.80020       1.00000  0.000000E+00       1.00000  0.000000E+00      0.798951       63.0225       1.00000       25.8712  0.000000E+00       3.52242  0.000000E+00       3.52242  0.000000E+00  0.000000E+00      -7.04485       44.9909       1.47392        1       Scatag_D
      55       1489.10       6807.86  0.000000E+00  0.000000E+00       2297.09  0.000000E+00       3256.09       2757.64  0.000000E+00       9623.91       37.6446       14998.3 -1.000000E+10 -1.000000E+10 -1.000000E+10 -1.000000E+10 -1.000000E+10  0.000000E+00  0.000000E+00  0.000000E+00  0.000000E+00  0.000000E+00  0.000000E+00  0.000000E+00  0.000000E+00  0.000000E+00  0.000000E+00  0.000000E+00  0.000000E+00       Funtag_D
      56  2.336143E+05  1.925924E+05       41021.9       71.7953      0.193437       1.72487      0.229209      0.241620    -0.0452292    -0.0637808    0.00731862       1.11491       Opttag_D
      56       45.3385       2.83900       1.00000  0.000000E+00       1.00000  0.000000E+00      0.799511       62.9513       1.00000       25.9500  0.000000E+00       3.50741  0.000000E+00       3.50741  0.000000E+00  0.000000E+00      -7.01482       44.9909       1.47392        1       Scatag_D
      56       1510.12       6784.16  0.000000E+00  0.000000E+00       2290.82  0.000000E+00       3219.88       2897.16  0.000000E+00       9462.19       37.3968       14820.2 -1.000000E+10 -1.000000E+10 -1.000000E+10 -1.000000E+10 -1.000000E+10  0.000000E+00  0.000000E+00  0.000000E+00  0.000000E+00  0.000000E+00  0.000000E+00  0.000000E+00  0.000000E+00  0.000000E+00  0.000000E+00  0.000000E+00  0.000000E+00       Funtag_D
      57  2.332376E+05  1.926097E+05       40627.9       23.6397      0.194049       1.73221      0.229235      0.241666    -0.0453338    -0.0639922    0.00727554       1.10638       Opttag_D
      57       45.3549       2.84335       1.00000  0.000000E+00       1.00000  0.000000E+00      0.799575       62.9417       1.00000       25.9591  0.000000E+00       3.50589  0.000000E+00       3.50589  0.000000E+00  0.000000E+00      -7.01178       44.9909       1.47392        1       Scatag_D
      57       1492.39       6680.71  0.000000E+00  0.000000E+00       2290.04  0.000000E+00       3214.18       2683.17  0.000000E+00       9443.15       37.3562       14786.9 -1.000000E+10 -1.000000E+10 -1.000000E+10 -1.000000E+10 -1.000000E+10  0.000000E+00  0.000000E+00  0.000000E+00  0.000000E+00  0.000000E+00  0.000000E+00  0.000000E+00  0.000000E+00  0.000000E+00  0.000000E+00  0.000000E+00  0.000000E+00       Funtag_D
      58  2.331068E+05  1.926154E+05       40491.4       16.3514      0.194327       1.73584      0.229242      0.241682    -0.0453684    -0.0640652    0.00723855       1.10501       Opttag_D
      58       45.3638       2.84549       1.00000  0.000000E+00       1.00000  0.000000E+00      0.799591       62.9427       1.00000       25.9621  0.000000E+00       3.50493  0.000000E+00       3.50493  0.000000E+00  0.000000E+00      -7.00986       44.9909       1.47392        1       Scatag_D
      58       1477.26       6664.20  0.000000E+00  0.000000E+00       2289.55  0.000000E+00       3209.14       2651.33  0.000000E+00       9430.46       37.4279       14732.0 -1.000000E+10 -1.000000E+10 -1.000000E+10 -1.000000E+10 -1.000000E+10  0.000000E+00  0.000000E+00  0.000000E+00  0.000000E+00  0.000000E+00  0.000000E+00  0.000000E+00  0.000000E+00  0.000000E+00  0.000000E+00  0.000000E+00  0.000000E+00       Funtag_D
      59  2.329226E+05  1.926673E+05       40255.3       21.3467      0.196260       1.75979      0.229318      0.241837    -0.0456822    -0.0646390    0.00720325       1.10231       Opttag_D
      59       45.4164       2.85953       1.00000  0.000000E+00       1.00000  0.000000E+00      0.799763       62.9269       1.00000       25.9892  0.000000E+00       3.49900  0.000000E+00       3.49900  0.000000E+00  0.000000E+00      -6.99801       44.9909       1.47392        1       Scatag_D
      59       1462.88       6631.73  0.000000E+00  0.000000E+00       2287.63  0.000000E+00       3192.14       2643.22  0.000000E+00       9371.77       37.3890       14628.6 -1.000000E+10 -1.000000E+10 -1.000000E+10 -1.000000E+10 -1.000000E+10  0.000000E+00  0.000000E+00  0.000000E+00  0.000000E+00  0.000000E+00  0.000000E+00  0.000000E+00  0.000000E+00  0.000000E+00  0.000000E+00  0.000000E+00  0.000000E+00       Funtag_D
      60  2.327224E+05  1.927411E+05       39981.3       39.9638      0.199393       1.79981      0.229424      0.242096    -0.0461288    -0.0654764    0.00717801       1.09928       Opttag_D
      60       45.5024       2.88145       1.00000  0.000000E+00       1.00000  0.000000E+00      0.800016       62.9047       1.00000       26.0361  0.000000E+00       3.48901  0.000000E+00       3.48901  0.000000E+00  0.000000E+00      -6.97801       44.9909       1.47392        1       Scatag_D
      60       1452.65       6595.27  0.000000E+00  0.000000E+00       2284.85  0.000000E+00       3160.79       2671.53  0.000000E+00       9281.54       36.7462       14497.9 -1.000000E+10 -1.000000E+10 -1.000000E+10 -1.000000E+10 -1.000000E+10  0.000000E+00  0.000000E+00  0.000000E+00  0.000000E+00  0.000000E+00  0.000000E+00  0.000000E+00  0.000000E+00  0.000000E+00  0.000000E+00  0.000000E+00  0.000000E+00       Funtag_D
      61  2.324907E+05  1.928292E+05       39661.6       23.3266      0.202702       1.84147      0.229560      0.242423    -0.0466617    -0.0664534    0.00716555       1.09361       Opttag_D
      61       45.5901       2.90508       1.00000  0.000000E+00       1.00000  0.000000E+00      0.800314       62.8717       1.00000       26.0874  0.000000E+00       3.47849  0.000000E+00       3.47849  0.000000E+00  0.000000E+00      -6.95699       44.9909       1.47392        1       Scatag_D
      61       1447.61       6527.42  0.000000E+00  0.000000E+00       2283.01  0.000000E+00       3130.36       2596.63  0.000000E+00       9199.88       36.5941       14440.1 -1.000000E+10 -1.000000E+10 -1.000000E+10 -1.000000E+10 -1.000000E+10  0.000000E+00  0.000000E+00  0.000000E+00  0.000000E+00  0.000000E+00  0.000000E+00  0.000000E+00  0.000000E+00  0.000000E+00  0.000000E+00  0.000000E+00  0.000000E+00       Funtag_D
      62  2.323116E+05  1.928822E+05       39429.5       17.6522      0.204883       1.86979      0.229639      0.242647    -0.0469825    -0.0670947    0.00716215       1.08939       Opttag_D
      62       45.6484       2.92017       1.00000  0.000000E+00       1.00000  0.000000E+00      0.800505       62.8464       1.00000       26.1237  0.000000E+00       3.47165  0.000000E+00       3.47165  0.000000E+00  0.000000E+00      -6.94331       44.9909       1.47392        1       Scatag_D
      62       1446.24       6477.08  0.000000E+00  0.000000E+00       2282.00  0.000000E+00       3102.67       2559.14  0.000000E+00       9147.50       36.6557       14378.2 -1.000000E+10 -1.000000E+10 -1.000000E+10 -1.000000E+10 -1.000000E+10  0.000000E+00  0.000000E+00  0.000000E+00  0.000000E+00  0.000000E+00  0.000000E+00  0.000000E+00  0.000000E+00  0.000000E+00  0.000000E+00  0.000000E+00  0.000000E+00       Funtag_D
      63  2.321165E+05  1.929652E+05       39151.3       26.6753      0.208164       1.91271      0.229761      0.242976    -0.0474849    -0.0680710    0.00718837       1.08305       Opttag_D
      63       45.7373       2.94370       1.00000  0.000000E+00       1.00000  0.000000E+00      0.800789       62.8147       1.00000       26.1775  0.000000E+00       3.46152  0.000000E+00       3.46152  0.000000E+00  0.000000E+00      -6.92304       44.9909       1.47392        1       Scatag_D
      63       1456.85       6401.95  0.000000E+00  0.000000E+00       2280.77  0.000000E+00       3055.25       2564.43  0.000000E+00       9072.82       37.1695       14282.1 -1.000000E+10 -1.000000E+10 -1.000000E+10 -1.000000E+10 -1.000000E+10  0.000000E+00  0.000000E+00  0.000000E+00  0.000000E+00  0.000000E+00  0.000000E+00  0.000000E+00  0.000000E+00  0.000000E+00  0.000000E+00  0.000000E+00  0.000000E+00       Funtag_D
      64  2.319466E+05  1.930467E+05       38899.9       27.6425      0.211261       1.95255      0.229891      0.243285    -0.0479783    -0.0690871    0.00721846       1.07652       Opttag_D
      64       45.8207       2.96626       1.00000  0.000000E+00       1.00000  0.000000E+00      0.801056       62.7832       1.00000       26.2316  0.000000E+00       3.45269  0.000000E+00       3.45269  0.000000E+00  0.000000E+00      -6.90538       44.9909       1.47392        1       Scatag_D
      64       1469.07       6324.98  0.000000E+00  0.000000E+00       2280.02  0.000000E+00       3008.78       2557.81  0.000000E+00       9008.80       37.8493       14212.6 -1.000000E+10 -1.000000E+10 -1.000000E+10 -1.000000E+10 -1.000000E+10  0.000000E+00  0.000000E+00  0.000000E+00  0.000000E+00  0.000000E+00  0.000000E+00  0.000000E+00  0.000000E+00  0.000000E+00  0.000000E+00  0.000000E+00  0.000000E+00       Funtag_D
      65  2.317795E+05  1.930778E+05       38701.7       19.9591      0.212449       1.96753      0.229935      0.243395    -0.0481663    -0.0694718    0.00721034       1.07250       Opttag_D
      65       45.8548       2.97541       1.00000  0.000000E+00       1.00000  0.000000E+00      0.801149       62.7762       1.00000       26.2509  0.000000E+00       3.44944  0.000000E+00       3.44944  0.000000E+00  0.000000E+00      -6.89887       44.9909       1.47392        1       Scatag_D
      65       1465.76       6277.83  0.000000E+00  0.000000E+00       2279.39  0.000000E+00       2984.62       2508.72  0.000000E+00       8982.64       38.0545       14164.7 -1.000000E+10 -1.000000E+10 -1.000000E+10 -1.000000E+10 -1.000000E+10  0.000000E+00  0.000000E+00  0.000000E+00  0.000000E+00  0.000000E+00  0.000000E+00  0.000000E+00  0.000000E+00  0.000000E+00  0.000000E+00  0.000000E+00  0.000000E+00       Funtag_D
      66  2.315438E+05  1.931522E+05       38391.5       21.3331      0.215214       2.00080      0.230043      0.243646    -0.0486169    -0.0704047    0.00718946       1.06541       Opttag_D
      66       45.9338       2.99726       1.00000  0.000000E+00       1.00000  0.000000E+00      0.801364       62.7667       1.00000       26.2952  0.000000E+00       3.44133  0.000000E+00       3.44133  0.000000E+00  0.000000E+00      -6.88267       44.9909       1.47392        1       Scatag_D
      66       1457.29       6195.06  0.000000E+00  0.000000E+00       2278.10  0.000000E+00       2932.36       2468.10  0.000000E+00       8927.42       37.9354       14095.3 -1.000000E+10 -1.000000E+10 -1.000000E+10 -1.000000E+10 -1.000000E+10  0.000000E+00  0.000000E+00  0.000000E+00  0.000000E+00  0.000000E+00  0.000000E+00  0.000000E+00  0.000000E+00  0.000000E+00  0.000000E+00  0.000000E+00  0.000000E+00       Funtag_D
      67  2.314139E+05  1.932235E+05       38190.4       30.5384      0.217923       2.03220      0.230150      0.243899    -0.0490482    -0.0712649    0.00715501       1.06034       Opttag_D
      67       46.0093       3.01824       1.00000  0.000000E+00       1.00000  0.000000E+00      0.801567       62.7576       1.00000       26.3396  0.000000E+00       3.43275  0.000000E+00       3.43275  0.000000E+00  0.000000E+00      -6.86550       44.9909       1.47392        1       Scatag_D
      67       1443.36       6136.25  0.000000E+00  0.000000E+00       2277.19  0.000000E+00       2885.22       2472.78  0.000000E+00       8880.75       37.2365       14057.6 -1.000000E+10 -1.000000E+10 -1.000000E+10 -1.000000E+10 -1.000000E+10  0.000000E+00  0.000000E+00  0.000000E+00  0.000000E+00  0.000000E+00  0.000000E+00  0.000000E+00  0.000000E+00  0.000000E+00  0.000000E+00  0.000000E+00  0.000000E+00       Funtag_D
      68  2.312679E+05  1.932462E+05       38021.7       18.8671      0.219010       2.04389      0.230187      0.244002    -0.0491852    -0.0715735    0.00711690       1.05763       Opttag_D
      68       46.0399       3.02614       1.00000  0.000000E+00       1.00000  0.000000E+00      0.801637       62.7535       1.00000       26.3589  0.000000E+00       3.42893  0.000000E+00       3.42893  0.000000E+00  0.000000E+00      -6.85786       44.9909       1.47392        1       Scatag_D
      68       1428.02       6104.98  0.000000E+00  0.000000E+00       2276.61  0.000000E+00       2864.18       2420.88  0.000000E+00       8862.03       36.6559       14028.4 -1.000000E+10 -1.000000E+10 -1.000000E+10 -1.000000E+10 -1.000000E+10  0.000000E+00  0.000000E+00  0.000000E+00  0.000000E+00  0.000000E+00  0.000000E+00  0.000000E+00  0.000000E+00  0.000000E+00  0.000000E+00  0.000000E+00  0.000000E+00       Funtag_D
      69  2.310835E+05  1.932985E+05       37785.0       16.2699      0.221469       2.07013      0.230270      0.244251    -0.0495019    -0.0722934    0.00704852       1.05338       Opttag_D
      69       46.1079       3.04388       1.00000  0.000000E+00       1.00000  0.000000E+00      0.801794       62.7436       1.00000       26.4032  0.000000E+00       3.42039  0.000000E+00       3.42039  0.000000E+00  0.000000E+00      -6.84078       44.9909       1.47392        1       Scatag_D
      69       1400.71       6055.96  0.000000E+00  0.000000E+00       2275.57  0.000000E+00       2816.22       2402.13  0.000000E+00       8824.34       35.7791       13974.3 -1.000000E+10 -1.000000E+10 -1.000000E+10 -1.000000E+10 -1.000000E+10  0.000000E+00  0.000000E+00  0.000000E+00  0.000000E+00  0.000000E+00  0.000000E+00  0.000000E+00  0.000000E+00  0.000000E+00  0.000000E+00  0.000000E+00  0.000000E+00       Funtag_D
      70  2.309612E+05  1.933454E+05       37615.8       20.6463      0.223562       2.09224      0.230345      0.244478    -0.0497857    -0.0729149    0.00700059       1.05037       Opttag_D
      70       46.1650       3.05901       1.00000  0.000000E+00       1.00000  0.000000E+00      0.801926       62.7316       1.00000       26.4420  0.000000E+00       3.41374  0.000000E+00       3.41374  0.000000E+00  0.000000E+00      -6.82748       44.9909       1.47392        1       Scatag_D
      70       1381.72       6021.46  0.000000E+00  0.000000E+00       2274.77  0.000000E+00       2769.02       2398.60  0.000000E+00       8797.05       35.6494       13937.6 -1.000000E+10 -1.000000E+10 -1.000000E+10 -1.000000E+10 -1.000000E+10  0.000000E+00  0.000000E+00  0.000000E+00  0.000000E+00  0.000000E+00  0.000000E+00  0.000000E+00  0.000000E+00  0.000000E+00  0.000000E+00  0.000000E+00  0.000000E+00       Funtag_D
      71  2.307688E+05  1.934457E+05       37323.2       19.8692      0.227669       2.13476      0.230508      0.244934    -0.0503926    -0.0742119    0.00692075       1.04568       Opttag_D
      71       46.2750       3.08917       1.00000  0.000000E+00       1.00000  0.000000E+00      0.802184       62.7057       1.00000       26.5194  0.000000E+00       3.40243  0.000000E+00       3.40243  0.000000E+00  0.000000E+00      -6.80487       44.9909       1.47392        1       Scatag_D
      71       1350.39       5967.77  0.000000E+00  0.000000E+00       2273.63  0.000000E+00       2672.45       2371.75  0.000000E+00       8758.13       35.9386       13893.1 -1.000000E+10 -1.000000E+10 -1.000000E+10 -1.000000E+10 -1.000000E+10  0.000000E+00  0.000000E+00  0.000000E+00  0.000000E+00  0.000000E+00  0.000000E+00  0.000000E+00  0.000000E+00  0.000000E+00  0.000000E+00  0.000000E+00  0.000000E+00       Funtag_D
      72  2.306287E+05  1.935232E+05       37105.5       29.9655      0.230626       2.16286      0.230632      0.245287    -0.0508617    -0.0752623    0.00686111       1.04284       Opttag_D
      72       46.3546       3.11162       1.00000  0.000000E+00       1.00000  0.000000E+00      0.802371       62.6834       1.00000       26.5744  0.000000E+00       3.39530  0.000000E+00       3.39530  0.000000E+00  0.000000E+00      -6.79061       44.9909       1.47392        1       Scatag_D
      72       1327.22       5935.40  0.000000E+00  0.000000E+00       2271.97  0.000000E+00       2588.51       2365.54  0.000000E+00       8739.07       36.4135       13841.4 -1.000000E+10 -1.000000E+10 -1.000000E+10 -1.000000E+10 -1.000000E+10  0.000000E+00  0.000000E+00  0.000000E+00  0.000000E+00  0.000000E+00  0.000000E+00  0.000000E+00  0.000000E+00  0.000000E+00  0.000000E+00  0.000000E+00  0.000000E+00       Funtag_D
      73  2.304738E+05  1.935448E+05       36929.0       17.0365      0.231414       2.16865      0.230665      0.245380    -0.0509927    -0.0755733    0.00681989       1.04019       Opttag_D
      73       46.3770       3.11781       1.00000  0.000000E+00       1.00000  0.000000E+00      0.802415       62.6762       1.00000       26.5870  0.000000E+00       3.39464  0.000000E+00       3.39464  0.000000E+00  0.000000E+00      -6.78929       44.9909       1.47392        1       Scatag_D
      73       1311.32       5905.27  0.000000E+00  0.000000E+00       2270.68  0.000000E+00       2555.04       2315.34  0.000000E+00       8734.37       36.7431       13800.2 -1.000000E+10 -1.000000E+10 -1.000000E+10 -1.000000E+10 -1.000000E+10  0.000000E+00  0.000000E+00  0.000000E+00  0.000000E+00  0.000000E+00  0.000000E+00  0.000000E+00  0.000000E+00  0.000000E+00  0.000000E+00  0.000000E+00  0.000000E+00       Funtag_D
      74  2.303415E+05  1.935622E+05       36779.4       15.3386      0.232089       2.17182      0.230687      0.245465    -0.0510976    -0.0758745    0.00678904       1.03784       Opttag_D
      74       46.3981       3.12337       1.00000  0.000000E+00       1.00000  0.000000E+00      0.802449       62.6715       1.00000       26.5945  0.000000E+00       3.39438  0.000000E+00       3.39438  0.000000E+00  0.000000E+00      -6.78876       44.9909       1.47392        1       Scatag_D
      74       1299.48       5878.58  0.000000E+00  0.000000E+00       2268.58  0.000000E+00       2520.25       2294.40  0.000000E+00       8730.35       36.8718       13750.9 -1.000000E+10 -1.000000E+10 -1.000000E+10 -1.000000E+10 -1.000000E+10  0.000000E+00  0.000000E+00  0.000000E+00  0.000000E+00  0.000000E+00  0.000000E+00  0.000000E+00  0.000000E+00  0.000000E+00  0.000000E+00  0.000000E+00  0.000000E+00       Funtag_D
      75  2.302087E+05  1.936011E+05       36607.6       21.5658      0.233878       2.18502      0.230739      0.245683    -0.0513330    -0.0765294    0.00671664       1.03543       Opttag_D
      75       46.4498       3.13696       1.00000  0.000000E+00       1.00000  0.000000E+00      0.802554       62.6636       1.00000       26.6216  0.000000E+00       3.39053  0.000000E+00       3.39053  0.000000E+00  0.000000E+00      -6.78105       44.9909       1.47392        1       Scatag_D
      75       1271.91       5851.33  0.000000E+00  0.000000E+00       2265.50  0.000000E+00       2467.04       2283.06  0.000000E+00       8728.98       36.7094       13703.0 -1.000000E+10 -1.000000E+10 -1.000000E+10 -1.000000E+10 -1.000000E+10  0.000000E+00  0.000000E+00  0.000000E+00  0.000000E+00  0.000000E+00  0.000000E+00  0.000000E+00  0.000000E+00  0.000000E+00  0.000000E+00  0.000000E+00  0.000000E+00       Funtag_D
      76  2.300513E+05  1.936586E+05       36392.7       21.0102      0.236725       2.20625      0.230819      0.246032    -0.0516812    -0.0774415    0.00659891       1.03220       Opttag_D
      76       46.5306       3.15796       1.00000  0.000000E+00       1.00000  0.000000E+00      0.802714       62.6563       1.00000       26.6638  0.000000E+00       3.38344  0.000000E+00       3.38344  0.000000E+00  0.000000E+00      -6.76688       44.9909       1.47392        1       Scatag_D
      76       1227.71       5814.97  0.000000E+00  0.000000E+00       2261.22  0.000000E+00       2398.58       2251.77  0.000000E+00       8742.91       36.1837       13659.4 -1.000000E+10 -1.000000E+10 -1.000000E+10 -1.000000E+10 -1.000000E+10  0.000000E+00  0.000000E+00  0.000000E+00  0.000000E+00  0.000000E+00  0.000000E+00  0.000000E+00  0.000000E+00  0.000000E+00  0.000000E+00  0.000000E+00  0.000000E+00       Funtag_D
      77  2.299207E+05  1.936741E+05       36246.5       16.8983      0.238119       2.21642      0.230835      0.246172    -0.0517751    -0.0777813    0.00651563       1.02932       Opttag_D
      77       46.5710       3.16719       1.00000  0.000000E+00       1.00000  0.000000E+00      0.802783       62.6553       1.00000       26.6853  0.000000E+00       3.37849  0.000000E+00       3.37849  0.000000E+00  0.000000E+00      -6.75698       44.9909       1.47392        1       Scatag_D
      77       1196.92       5782.48  0.000000E+00  0.000000E+00       2258.97  0.000000E+00       2374.72       2229.32  0.000000E+00       8753.19       35.5784       13615.3 -1.000000E+10 -1.000000E+10 -1.000000E+10 -1.000000E+10 -1.000000E+10  0.000000E+00  0.000000E+00  0.000000E+00  0.000000E+00  0.000000E+00  0.000000E+00  0.000000E+00  0.000000E+00  0.000000E+00  0.000000E+00  0.000000E+00  0.000000E+00       Funtag_D
      78  2.297820E+05  1.936818E+05       36100.2       16.3057      0.239754       2.22755      0.230828      0.246327    -0.0518214    -0.0781491    0.00642187       1.02586       Opttag_D
      78       46.6195       3.17720       1.00000  0.000000E+00       1.00000  0.000000E+00      0.802849       62.6569       1.00000       26.7103  0.000000E+00       3.37160  0.000000E+00       3.37160  0.000000E+00  0.000000E+00      -6.74320       44.9909       1.47392        1       Scatag_D
      78       1162.72       5743.69  0.000000E+00  0.000000E+00       2255.99  0.000000E+00       2353.99       2226.04  0.000000E+00       8767.81       34.8651       13555.1 -1.000000E+10 -1.000000E+10 -1.000000E+10 -1.000000E+10 -1.000000E+10  0.000000E+00  0.000000E+00  0.000000E+00  0.000000E+00  0.000000E+00  0.000000E+00  0.000000E+00  0.000000E+00  0.000000E+00  0.000000E+00  0.000000E+00  0.000000E+00       Funtag_D
      79  2.296817E+05  1.936813E+05       36000.4       18.6050      0.240950       2.23420      0.230810      0.246404    -0.0518185    -0.0783264    0.00635082       1.02325       Opttag_D
      79       46.6561       3.18399       1.00000  0.000000E+00       1.00000  0.000000E+00      0.802886       62.6576       1.00000       26.7275  0.000000E+00       3.36685  0.000000E+00       3.36685  0.000000E+00  0.000000E+00      -6.73371       44.9909       1.47392        1       Scatag_D
      79       1137.13       5714.52  0.000000E+00  0.000000E+00       2253.20  0.000000E+00       2341.06       2232.27  0.000000E+00       8780.16       34.5845       13507.5 -1.000000E+10 -1.000000E+10 -1.000000E+10 -1.000000E+10 -1.000000E+10  0.000000E+00  0.000000E+00  0.000000E+00  0.000000E+00  0.000000E+00  0.000000E+00  0.000000E+00  0.000000E+00  0.000000E+00  0.000000E+00  0.000000E+00  0.000000E+00       Funtag_D
      80  2.295675E+05  1.936775E+05       35890.0       13.7100      0.242008       2.23891      0.230783      0.246470    -0.0517953    -0.0785335    0.00628229       1.02094       Opttag_D
      80       46.6895       3.18976       1.00000  0.000000E+00       1.00000  0.000000E+00      0.802911       62.6572       1.00000       26.7434  0.000000E+00       3.36306  0.000000E+00       3.36306  0.000000E+00  0.000000E+00      -6.72611       44.9909       1.47392        1       Scatag_D
      80       1112.73       5688.78  0.000000E+00  0.000000E+00       2250.26  0.000000E+00       2329.19       2217.61  0.000000E+00       8792.44       34.6041       13464.4 -1.000000E+10 -1.000000E+10 -1.000000E+10 -1.000000E+10 -1.000000E+10  0.000000E+00  0.000000E+00  0.000000E+00  0.000000E+00  0.000000E+00  0.000000E+00  0.000000E+00  0.000000E+00  0.000000E+00  0.000000E+00  0.000000E+00  0.000000E+00       Funtag_D
      81  2.294508E+05  1.936822E+05       35768.6       16.5603      0.243496       2.24498      0.230768      0.246576    -0.0518240    -0.0789169    0.00619339       1.01899       Opttag_D
      81       46.7361       3.19883       1.00000  0.000000E+00       1.00000  0.000000E+00      0.802963       62.6534       1.00000       26.7658  0.000000E+00       3.35889  0.000000E+00       3.35889  0.000000E+00  0.000000E+00      -6.71779       44.9909       1.47392        1       Scatag_D
      81       1081.46       5667.05  0.000000E+00  0.000000E+00       2245.94  0.000000E+00       2305.83       2208.70  0.000000E+00       8814.26       35.0462       13410.3 -1.000000E+10 -1.000000E+10 -1.000000E+10 -1.000000E+10 -1.000000E+10  0.000000E+00  0.000000E+00  0.000000E+00  0.000000E+00  0.000000E+00  0.000000E+00  0.000000E+00  0.000000E+00  0.000000E+00  0.000000E+00  0.000000E+00  0.000000E+00       Funtag_D
      82  2.293690E+05  1.936892E+05       35679.8       19.3612      0.244661       2.26791      0.230762      0.246668    -0.0518664    -0.0792513    0.00613576       1.01764       Opttag_D
      82       46.7730       3.20640       1.00000  0.000000E+00       1.00000  0.000000E+00      0.803014       62.6517       1.00000       26.7823  0.000000E+00       3.35666  0.000000E+00       3.35666  0.000000E+00  0.000000E+00      -6.71333       44.9909       1.47392        1       Scatag_D
      82       1061.42       5652.01  0.000000E+00  0.000000E+00       2242.40  0.000000E+00       2288.09       2203.80  0.000000E+00       8831.39       35.4452       13365.2 -1.000000E+10 -1.000000E+10 -1.000000E+10 -1.000000E+10 -1.000000E+10  0.000000E+00  0.000000E+00  0.000000E+00  0.000000E+00  0.000000E+00  0.000000E+00  0.000000E+00  0.000000E+00  0.000000E+00  0.000000E+00  0.000000E+00  0.000000E+00       Funtag_D
      83  2.292992E+05  1.936910E+05       35608.2       12.8495      0.245156       2.28479      0.230759      0.246708    -0.0518769    -0.0793926    0.00610847       1.01691       Opttag_D
      83       46.7893       3.20962       1.00000  0.000000E+00       1.00000  0.000000E+00      0.803039       62.6513       1.00000       26.7891  0.000000E+00       3.35554  0.000000E+00       3.35554  0.000000E+00  0.000000E+00      -6.71109       44.9909       1.47392        1       Scatag_D
      83       1052.00       5643.94  0.000000E+00  0.000000E+00       2240.68  0.000000E+00       2281.14       2182.51  0.000000E+00       8835.04       35.5408       13337.4 -1.000000E+10 -1.000000E+10 -1.000000E+10 -1.000000E+10 -1.000000E+10  0.000000E+00  0.000000E+00  0.000000E+00  0.000000E+00  0.000000E+00  0.000000E+00  0.000000E+00  0.000000E+00  0.000000E+00  0.000000E+00  0.000000E+00  0.000000E+00       Funtag_D
      84  2.292177E+05  1.936888E+05       35529.0       10.9468      0.245998       2.31219      0.230742      0.246778    -0.0518635    -0.0796066    0.00606817       1.01591       Opttag_D
      84       46.8171       3.21462       1.00000  0.000000E+00       1.00000  0.000000E+00      0.803079       62.6532       1.00000       26.8003  0.000000E+00       3.35274  0.000000E+00       3.35274  0.000000E+00  0.000000E+00      -6.70548       44.9909       1.47392        1       Scatag_D
      84       1038.17       5632.79  0.000000E+00  0.000000E+00       2237.90  0.000000E+00       2274.59       2175.32  0.000000E+00       8840.24       35.3828       13294.6 -1.000000E+10 -1.000000E+10 -1.000000E+10 -1.000000E+10 -1.000000E+10  0.000000E+00  0.000000E+00  0.000000E+00  0.000000E+00  0.000000E+00  0.000000E+00  0.000000E+00  0.000000E+00  0.000000E+00  0.000000E+00  0.000000E+00  0.000000E+00       Funtag_D
      85  2.291426E+05  1.936761E+05       35466.6       20.2687      0.247552       2.35869      0.230698      0.246860    -0.0517867    -0.0798655    0.00598936       1.01498       Opttag_D
      85       46.8660       3.22247       1.00000  0.000000E+00       1.00000  0.000000E+00      0.803136       62.6565       1.00000       26.8237  0.000000E+00       3.34696  0.000000E+00       3.34696  0.000000E+00  0.000000E+00      -6.69391       44.9909       1.47392        1       Scatag_D
      85       1011.38       5622.47  0.000000E+00  0.000000E+00       2233.54  0.000000E+00       2270.44       2198.81  0.000000E+00       8857.80       34.8149       13237.3 -1.000000E+10 -1.000000E+10 -1.000000E+10 -1.000000E+10 -1.000000E+10  0.000000E+00  0.000000E+00  0.000000E+00  0.000000E+00  0.000000E+00  0.000000E+00  0.000000E+00  0.000000E+00  0.000000E+00  0.000000E+00  0.000000E+00  0.000000E+00       Funtag_D
      86  2.290578E+05  1.936613E+05       35396.5       13.7687      0.248937       2.39849      0.230652      0.246943    -0.0516976    -0.0801100    0.00592370       1.01482       Opttag_D
      86       46.9091       3.22900       1.00000  0.000000E+00       1.00000  0.000000E+00      0.803184       62.6611       1.00000       26.8455  0.000000E+00       3.34144  0.000000E+00       3.34144  0.000000E+00  0.000000E+00      -6.68289       44.9909       1.47392        1       Scatag_D
      86       989.325       5620.74  0.000000E+00  0.000000E+00       2229.92  0.000000E+00       2271.38       2189.79  0.000000E+00       8871.63       34.2658       13189.4 -1.000000E+10 -1.000000E+10 -1.000000E+10 -1.000000E+10 -1.000000E+10  0.000000E+00  0.000000E+00  0.000000E+00  0.000000E+00  0.000000E+00  0.000000E+00  0.000000E+00  0.000000E+00  0.000000E+00  0.000000E+00  0.000000E+00  0.000000E+00       Funtag_D
      87  2.289874E+05  1.936458E+05       35341.6       11.9282      0.249938       2.42546      0.230613      0.246980    -0.0516036    -0.0802350    0.00586850       1.01494       Opttag_D
      87       46.9399       3.23309       1.00000  0.000000E+00       1.00000  0.000000E+00      0.803212       62.6636       1.00000       26.8627  0.000000E+00       3.33726  0.000000E+00       3.33726  0.000000E+00  0.000000E+00      -6.67453       44.9909       1.47392        1       Scatag_D
      87       970.973       5622.10  0.000000E+00  0.000000E+00       2227.21  0.000000E+00       2273.37       2194.05  0.000000E+00       8875.39       34.0042       13144.5 -1.000000E+10 -1.000000E+10 -1.000000E+10 -1.000000E+10 -1.000000E+10  0.000000E+00  0.000000E+00  0.000000E+00  0.000000E+00  0.000000E+00  0.000000E+00  0.000000E+00  0.000000E+00  0.000000E+00  0.000000E+00  0.000000E+00  0.000000E+00       Funtag_D

 An atom has moved more than     1.50 A from the initial/last
 position - if this was an accepted trial point, probably need to update the
 non-bonded contact list.

      88  2.289002E+05  1.936260E+05       35274.2       15.3133      0.251487       2.46549      0.230559      0.247039    -0.0514835    -0.0804749    0.00580557       1.01619       Opttag_D
      88       46.9872       3.23970       1.00000  0.000000E+00       1.00000  0.000000E+00      0.803264       62.6657       1.00000       26.8909  0.000000E+00       3.33182  0.000000E+00       3.33182  0.000000E+00  0.000000E+00      -6.66363       44.9909       1.47392        1       Scatag_D
      88       950.259       5635.95  0.000000E+00  0.000000E+00       2223.01  0.000000E+00       2275.56       2214.04  0.000000E+00       8874.44       34.0258       13067.0 -1.000000E+10 -1.000000E+10 -1.000000E+10 -1.000000E+10 -1.000000E+10  0.000000E+00  0.000000E+00  0.000000E+00  0.000000E+00  0.000000E+00  0.000000E+00  0.000000E+00  0.000000E+00  0.000000E+00  0.000000E+00  0.000000E+00  0.000000E+00       Funtag_D

 An atom has moved more than     1.50 A from the initial/last
 position - need to update the non-bonded contact list.


 ############################################################################################################
 ###################################################### setting up contact terms
 ############################################################################################################

      89  2.288153E+05  1.936096E+05       35205.7       17.1263      0.253144       2.50534      0.230514      0.247099    -0.0513843    -0.0807191    0.00576746       1.01858       Opttag_D
      89       47.0371       3.24706       1.00000  0.000000E+00       1.00000  0.000000E+00      0.803331       62.6666       1.00000       26.9230  0.000000E+00       3.32677  0.000000E+00       3.32677  0.000000E+00  0.000000E+00      -6.65353       44.9909       1.47392        1       Scatag_D
      89       937.827       5662.43  0.000000E+00  0.000000E+00       2218.64  0.000000E+00       2278.06       2231.95  0.000000E+00       8862.95       34.3776       12979.5 -1.000000E+10 -1.000000E+10 -1.000000E+10 -1.000000E+10 -1.000000E+10  0.000000E+00  0.000000E+00  0.000000E+00  0.000000E+00  0.000000E+00  0.000000E+00  0.000000E+00  0.000000E+00  0.000000E+00  0.000000E+00  0.000000E+00  0.000000E+00       Funtag_D
      90  2.287180E+05  1.935986E+05       35119.4       16.2933      0.253550       2.51162      0.230490      0.247106    -0.0513178    -0.0807932    0.00577529       1.01950       Opttag_D
      90       47.0514       3.24825       1.00000  0.000000E+00       1.00000  0.000000E+00      0.803352       62.6613       1.00000       26.9351  0.000000E+00       3.32603  0.000000E+00       3.32603  0.000000E+00  0.000000E+00      -6.65206       44.9909       1.47392        1       Scatag_D
      90       940.375       5672.70  0.000000E+00  0.000000E+00       2216.28  0.000000E+00       2273.87       2225.87  0.000000E+00       8826.86       34.8703       12928.5 -1.000000E+10 -1.000000E+10 -1.000000E+10 -1.000000E+10 -1.000000E+10  0.000000E+00  0.000000E+00  0.000000E+00  0.000000E+00  0.000000E+00  0.000000E+00  0.000000E+00  0.000000E+00  0.000000E+00  0.000000E+00  0.000000E+00  0.000000E+00       Funtag_D
      91  2.286100E+05  1.935901E+05       35019.8       14.9324      0.253336       2.50209      0.230480      0.247081    -0.0512668    -0.0807615    0.00580781       1.01943       Opttag_D
      91       47.0486       3.24644       1.00000  0.000000E+00       1.00000  0.000000E+00      0.803351       62.6554       1.00000       26.9363  0.000000E+00       3.32769  0.000000E+00       3.32769  0.000000E+00  0.000000E+00      -6.65538       44.9909       1.47392        1       Scatag_D
      91       950.995       5671.90  0.000000E+00  0.000000E+00       2214.79  0.000000E+00       2265.35       2215.10  0.000000E+00       8778.40       35.1359       12888.2 -1.000000E+10 -1.000000E+10 -1.000000E+10 -1.000000E+10 -1.000000E+10  0.000000E+00  0.000000E+00  0.000000E+00  0.000000E+00  0.000000E+00  0.000000E+00  0.000000E+00  0.000000E+00  0.000000E+00  0.000000E+00  0.000000E+00  0.000000E+00       Funtag_D
      92  2.285213E+05  1.935701E+05       34951.1       20.5080      0.253451       2.50065      0.230444      0.247079    -0.0511456    -0.0807517    0.00583169       1.01995       Opttag_D
      92       47.0552       3.24499       1.00000  0.000000E+00       1.00000  0.000000E+00      0.803359       62.6489       1.00000       26.9463  0.000000E+00       3.32760  0.000000E+00       3.32760  0.000000E+00  0.000000E+00      -6.65519       44.9909       1.47392        1       Scatag_D
      92       958.831       5677.69  0.000000E+00  0.000000E+00       2212.71  0.000000E+00       2262.79       2232.96  0.000000E+00       8727.37       34.9551       12843.8 -1.000000E+10 -1.000000E+10 -1.000000E+10 -1.000000E+10 -1.000000E+10  0.000000E+00  0.000000E+00  0.000000E+00  0.000000E+00  0.000000E+00  0.000000E+00  0.000000E+00  0.000000E+00  0.000000E+00  0.000000E+00  0.000000E+00  0.000000E+00       Funtag_D
      93  2.284204E+05  1.935489E+05       34871.5       13.7648      0.253678       2.50347      0.230410      0.247070    -0.0510171    -0.0806993    0.00583312       1.01986       Opttag_D
      93       47.0633       3.24373       1.00000  0.000000E+00       1.00000  0.000000E+00      0.803368       62.6430       1.00000       26.9577  0.000000E+00       3.32670  0.000000E+00       3.32670  0.000000E+00  0.000000E+00      -6.65339       44.9909       1.47392        1       Scatag_D
      93       959.299       5676.70  0.000000E+00  0.000000E+00       2211.28  0.000000E+00       2265.31       2220.34  0.000000E+00       8694.83       34.5228       12809.2 -1.000000E+10 -1.000000E+10 -1.000000E+10 -1.000000E+10 -1.000000E+10  0.000000E+00  0.000000E+00  0.000000E+00  0.000000E+00  0.000000E+00  0.000000E+00  0.000000E+00  0.000000E+00  0.000000E+00  0.000000E+00  0.000000E+00  0.000000E+00       Funtag_D
      94  2.282946E+05  1.935077E+05       34786.9       15.1882      0.254635       2.52229      0.230336      0.247082    -0.0507680    -0.0806774    0.00580950       1.02013       Opttag_D
      94       47.0908       3.24360       1.00000  0.000000E+00       1.00000  0.000000E+00      0.803402       62.6344       1.00000       26.9885  0.000000E+00       3.32304  0.000000E+00       3.32304  0.000000E+00  0.000000E+00      -6.64607       44.9909       1.47392        1       Scatag_D
      94       951.545       5679.68  0.000000E+00  0.000000E+00       2208.83  0.000000E+00       2277.91       2231.08  0.000000E+00       8657.05       33.7485       12747.1 -1.000000E+10 -1.000000E+10 -1.000000E+10 -1.000000E+10 -1.000000E+10  0.000000E+00  0.000000E+00  0.000000E+00  0.000000E+00  0.000000E+00  0.000000E+00  0.000000E+00  0.000000E+00  0.000000E+00  0.000000E+00  0.000000E+00  0.000000E+00       Funtag_D
      95  2.282070E+05  1.934622E+05       34744.8       24.9069      0.255879       2.54176      0.230255      0.247102    -0.0504923    -0.0806851    0.00584162       1.02116       Opttag_D
      95       47.1251       3.24387       1.00000  0.000000E+00       1.00000  0.000000E+00      0.803448       62.6192       1.00000       27.0308  0.000000E+00       3.31932  0.000000E+00       3.31932  0.000000E+00  0.000000E+00      -6.63864       44.9909       1.47392        1       Scatag_D
      95       962.099       5691.14  0.000000E+00  0.000000E+00       2206.05  0.000000E+00       2289.54       2267.39  0.000000E+00       8610.47       33.6196       12684.5 -1.000000E+10 -1.000000E+10 -1.000000E+10 -1.000000E+10 -1.000000E+10  0.000000E+00  0.000000E+00  0.000000E+00  0.000000E+00  0.000000E+00  0.000000E+00  0.000000E+00  0.000000E+00  0.000000E+00  0.000000E+00  0.000000E+00  0.000000E+00       Funtag_D
      96  2.281058E+05  1.934487E+05       34657.2       13.8254      0.256206       2.54528      0.230230      0.247107    -0.0504105    -0.0806943    0.00584051       1.02024       Opttag_D
      96       47.1337       3.24356       1.00000  0.000000E+00       1.00000  0.000000E+00      0.803452       62.6112       1.00000       27.0440  0.000000E+00       3.31840  0.000000E+00       3.31840  0.000000E+00  0.000000E+00      -6.63680       44.9909       1.47392        1       Scatag_D
      96       961.731       5680.92  0.000000E+00  0.000000E+00       2205.41  0.000000E+00       2288.65       2238.77  0.000000E+00       8599.54       33.7763       12648.4 -1.000000E+10 -1.000000E+10 -1.000000E+10 -1.000000E+10 -1.000000E+10  0.000000E+00  0.000000E+00  0.000000E+00  0.000000E+00  0.000000E+00  0.000000E+00  0.000000E+00  0.000000E+00  0.000000E+00  0.000000E+00  0.000000E+00  0.000000E+00       Funtag_D
      97  2.280244E+05  1.934467E+05       34577.7       11.3490      0.256159       2.54109      0.230225      0.247103    -0.0503988    -0.0806949    0.00584881       1.01914       Opttag_D
      97       47.1328       3.24289       1.00000  0.000000E+00       1.00000  0.000000E+00      0.803436       62.6050       1.00000       27.0461  0.000000E+00       3.31896  0.000000E+00       3.31896  0.000000E+00  0.000000E+00      -6.63791       44.9909       1.47392        1       Scatag_D
      97       964.468       5668.68  0.000000E+00  0.000000E+00       2205.26  0.000000E+00       2278.22       2235.20  0.000000E+00       8595.86       34.2623       12595.8 -1.000000E+10 -1.000000E+10 -1.000000E+10 -1.000000E+10 -1.000000E+10  0.000000E+00  0.000000E+00  0.000000E+00  0.000000E+00  0.000000E+00  0.000000E+00  0.000000E+00  0.000000E+00  0.000000E+00  0.000000E+00  0.000000E+00  0.000000E+00       Funtag_D
      98  2.279428E+05  1.934262E+05       34516.6       16.6588      0.256473       2.54176      0.230196      0.247109    -0.0502747    -0.0806981    0.00586368       1.01934       Opttag_D
      98       47.1417       3.24144       1.00000  0.000000E+00       1.00000  0.000000E+00      0.803432       62.5896       1.00000       27.0647  0.000000E+00       3.31859  0.000000E+00       3.31859  0.000000E+00  0.000000E+00      -6.63719       44.9909       1.47392        1       Scatag_D
      98       969.379       5670.92  0.000000E+00  0.000000E+00       2204.28  0.000000E+00       2273.69       2249.00  0.000000E+00       8573.96       34.1796       12541.2 -1.000000E+10 -1.000000E+10 -1.000000E+10 -1.000000E+10 -1.000000E+10  0.000000E+00  0.000000E+00  0.000000E+00  0.000000E+00  0.000000E+00  0.000000E+00  0.000000E+00  0.000000E+00  0.000000E+00  0.000000E+00  0.000000E+00  0.000000E+00       Funtag_D
      99  2.278357E+05  1.933934E+05       34442.3       15.9238      0.256795       2.53601      0.230150      0.247103    -0.0500764    -0.0806595    0.00592711       1.02039       Opttag_D
      99       47.1510       3.23794       1.00000  0.000000E+00       1.00000  0.000000E+00      0.803409       62.5577       1.00000       27.0938  0.000000E+00       3.31893  0.000000E+00       3.31893  0.000000E+00  0.000000E+00      -6.63786       44.9909       1.47392        1       Scatag_D
      99       990.463       5682.67  0.000000E+00  0.000000E+00       2202.94  0.000000E+00       2262.63       2253.72  0.000000E+00       8542.32       34.1389       12473.4 -1.000000E+10 -1.000000E+10 -1.000000E+10 -1.000000E+10 -1.000000E+10  0.000000E+00  0.000000E+00  0.000000E+00  0.000000E+00  0.000000E+00  0.000000E+00  0.000000E+00  0.000000E+00  0.000000E+00  0.000000E+00  0.000000E+00  0.000000E+00       Funtag_D

   MaxLik Scaling Cycle 0104

   Wilson plot

   Statistics in resolution bins after refinement cycle 0003
```


|  |  |
| --- | --- |
|  | - Rxpct factors plot- Rxpct factors table- <Log-Lik Gain>- Ln Sigma A- Correlation Coefficients |


```
   Iters     Tot_Funct    Xray_Funct    Geom_Funct          Grms     rmsD2init     maxD2init             R         Rfree           LLG       LLGfree     rms(bond)    rms(angle)
   Iters             K   B_IMPF_FRAG        K_MISS   B_TEMP_MISS   K_IMPF_MISS   B_IMPF_MISS        K_SOLV   B_TEMP_SOLV   K_IMPF_SOLV   B_IMPF_SOLV        B_TEMP     B_TEMP_11     B_TEMP_12     B_TEMP_22     B_TEMP_13     B_TEMP_23     B_TEMP_33      K_WILSON      B_WILSON      FGCYCLES
   Iters          BOND         ANGLE      IMPROPER       TORSION    SINTORSION      TRIGONAL        PLANAR  BCORRELATION       CONTACT     IDEALDIST        CHIRAL    SIMILARITY       OCCSSUM  UTILDISTANCE     UTILANGLE   UTILTORSION        RHOFIT        ADP-01        ADP-02        ADP-03        ADP-04        ADP-05        ADP-06        ADP-07        ADP-08        ADP-09        ADP-10      RHOFITPC            QM
     100  2.277499E+05  1.933717E+05       34378.1       16.4771      0.256885       2.53002      0.230119      0.247101    -0.0499451    -0.0806275    0.00592692       1.01971       Opttag_D
     100       47.1531       3.23513       1.00000  0.000000E+00       1.00000  0.000000E+00      0.803382       62.5425       1.00000       27.1065  0.000000E+00       3.31889  0.000000E+00       3.31889  0.000000E+00  0.000000E+00      -6.63778       44.9909       1.47392        1       Scatag_D
     100       990.401       5675.04  0.000000E+00  0.000000E+00       2202.52  0.000000E+00       2263.35       2247.88  0.000000E+00       8530.30       33.8057       12434.9 -1.000000E+10 -1.000000E+10 -1.000000E+10 -1.000000E+10 -1.000000E+10  0.000000E+00  0.000000E+00  0.000000E+00  0.000000E+00  0.000000E+00  0.000000E+00  0.000000E+00  0.000000E+00  0.000000E+00  0.000000E+00  0.000000E+00  0.000000E+00       Funtag_D
   Geometry Restraint Deviations SCREEN Information

 
 No -bcorrel_zeroocchydrogens_factor flag found - keeping default: will set B for OCC=0 hydrogens to    1.200 times heavy-atom B


 TLS information. Total number of groups:     2


 Group   Natoms    FixRB   FixTLS   Group Tag    Group Spec
     1     2785      Y        Y     tls1    { A|* }
     2     2772      Y        Y     tls2    { B|* }
 
NOTE BUSTER_TLS_SET tls1 { A|* }
NOTE BUSTER_TLS_T   tls1     -1.04460E-01 -3.29576E-03  1.26753E-01 -2.05452E-02  1.97227E-02  9.29085E-03
NOTE BUSTER_TLS_L   tls1      9.04625E-01  1.02472E+00  1.05382E-01  2.72562E-01 -2.87417E-01  3.46645E-01
NOTE BUSTER_TLS_S   tls1      2.27101E-01 -2.03443E-01  2.22735E-01 -5.71643E-02 -4.35115E-03 -1.74217E-01 -1.22766E-01 -1.55520E-02
NOTE BUSTER_TLS_O   tls1     -2.33111E+01 -8.37601E+01  1.13266E+01
NOTE BUSTER_TLS_FIX tls1 ALL
 
NOTE BUSTER_TLS_SET tls2 { B|* }
NOTE BUSTER_TLS_T   tls2     -1.36395E-01 -9.35808E-02  2.57140E-01  1.23450E-02  4.34122E-02  4.18694E-03
NOTE BUSTER_TLS_L   tls2      6.37772E-01  2.55963E-01  4.61831E-01  3.69121E-01 -3.49623E-01 -2.69037E-01
NOTE BUSTER_TLS_S   tls2     -7.13175E-03  7.18486E-02 -5.60545E-02  8.36950E-02  1.18915E-01 -1.98565E-02 -1.56302E-02  2.94633E-02
NOTE BUSTER_TLS_O   tls2      2.21121E+01 -7.20987E+01  3.46915E+00
NOTE BUSTER_TLS_FIX tls2 ALL

 For final position, Geometry function summary
     term                           number     weight       rms        GooF      function     fn/numb
     ================================================================================================
     BOND bond lengths (angs)       5676       2.000      0.0059       0.295       990.40       0.174
     ANGL bond angles (degs)        7707       2.000        1.02       0.607      5675.04       0.736
     TORS split-harmonic tors       1281       0.000       16.89       1.059         0.00       0.000
     SINTOR sinusoidal tors         1281       2.000       16.89                  2202.52       1.719
     PLAN general plane (angs)       966       5.000      0.0137       0.685      2263.35       2.343
     BCORrelations (angs^2)         5676      10.000       0.557       0.199      2247.88       0.396
     CONT (bad contacts)               0       5.000                                 0.00
     IDEAL(ideal-dist contact)      5010       4.000                              8530.30
     CHIRAL (gelly semiharmon)       740       5.000                                33.81
     SIM similarity restraint for NCS and/or TARGET                              12434.85
     OCCSUM restraint on sum of occupanies                                           0.00       as none defined
     DISTAN utility restraints interatomic distance                                  0.00       as none defined
     UTILANGLE utility angle restraints                                              0.00       as none defined
     UTILTOR utility restraints on torsion angles                                    0.00       as none defined
     Peptide omega torsion angles                           2.60 degs - monitor only
 
 WARNING: Have |delta/sigma| deviations > 5.0 sigma. Number of outliers for each term: 
 WARNING:         2 bond lengths. Worst is     6.8 sigs     1.25 Angs A|501:C05=C04 (LIG)
 WARNING:         5 bond angles.  Worst is    21.9 sigs    54.76 degs A|501:C09=C04=C05 (LIG)
 WARNING:         1 planes.       Worst is     9.4 sigs     0.19 Angs A|501:N03=C04=C05=C06=C07=C08=C09=C10 (LIG)
 WARNING:         1 idealD contacts. Worst     8.3 sigs     2.01 Angs A|501:C09=C11 (LIG)

 Weighted rms      0.0059         1.020  (temporary rms bond and angle for BUSTER to pick up!)
 Weighted intra-residual rms(bond) for StdProtein (excluding H)      0.0051
 Weighted intra-residual rms(bond) for Buffer     (excluding H)      0.0037
 Outputing geometry progress graph in plotmtv format to file:Geom_Res.mtv

 ############### Running final geometry/screen analysis (screen_final.txt)

 s/r gelly normal termination
```

Statistics and scale factors vs. cycle number
```

|  |  |  |
| --- | --- | --- |
|  | - Rxpct factors- <Log-Lik Gain>- GEOM Residuals- Ln Sigma_A_ | - Scale Factors- B Factors- Imperfection Factors |

```
   Final Geometry Information


---


### 2. Final results

Statistics and scale factors vs. cycle number
```

|  |  |  |
| --- | --- | --- |
|  | - Rxpct factors- <Log-Lik Gain>- GEOM Residuals- Ln Sigma_A_ | - Scale Factors- B Factors- Imperfection Factors |

```
   Average Figure of Merit
   Structure Factor Amplitudes


 # normal termination

  Program stopped.
```

closesok message: time spent in tree = 1796.59 (user) 37.93 (sys)

---

# Normal termination

```
 Resources usage summary:
     Time spent in client ...................  1796.60 (user)       37.95 (system)
     Time spent in scripts (    27 calls) ...     3.10 (user)        0.19 (system)
     Time spent in server ...................     0.00 (user)        0.00 (system)

     Max resident memory used (kb) .......... 469152
     Number of page faults .................. 0 (major)    1414785 (minor)
     Number of context switches ............. 4259 (niced)    6742 (other)
```
